# Single‐cell transcriptome atlas reveals a *WRKY33* ‐ *HSP81* regulatory module that orchestrates phenylpropanoid‐mediated defense against *Sclerotium rolfsii* in *Dendrobium officinale*

**DOI:** 10.1111/tpj.71190

**Published:** 2026-10-29

**Authors:** Lingli Li, Ye Xu, Wei Liu, Zhitao Niu, Qingyun Xue, Xiaoyu Ding

**Affiliations:** ^1^ College of Life Sciences, Nanjing Normal University Nanjing 210023 China; ^2^ Jiangsu Provincial Engineering Research Center for Technical Industrialization of Dendrobiums Nanjing 210023 China

**Keywords:** single‐cell RNA sequencing, *Dendrobium officinale*, *Sclerotium rolfsii*, *WRKY33*‐*HSP81* regulatory module, phenylpropanoid biosynthesis pathway

## Abstract

Plants employ cell‐type‐specific transcriptional programs to defend against pathogens, yet the cellular heterogeneity of immune responses in the medicinal orchid *Dendrobium officinale* Kimura et Migo remains largely unexplored. A single‐cell transcriptomic atlas of the *D. officinale* stem base upon *Sclerotium rolfsii* infection was constructed, profiling over 20 000 cells across 12 and 24 h post‐infection. A total of 21 cell clusters were resolved into 6 major cell types—epidermal, ground tissue parenchyma, proliferating, xylem, phloem, and vascular bundle parenchyma cells—with identities validated by fluorescence *in situ* hybridization. Cell‐type‐specific analyses revealed that epidermal and phloem cells mounted early defense responses, while xylem cells exhibited the most extensive transcriptional reprogramming at 24 hpi, coinciding with the activation of lignin biosynthesis pathways. *WRKY33* was identified as a central transcriptional activator that directly binds W‐box motifs in the promoters of lignin biosynthetic genes *C4H* and *HCT*, promoting lignin deposition and enhancing disease resistance. Conversely, the nuclear‐localized chaperone *HSP81* physically interacts with *WRKY33* and functions as a broad regulator of multiple transcription factors via co‐factor sequestration, thereby compromising defense when overexpressed. These findings uncover a *WRKY33*‐*HSP81* regulatory module that orchestrates phenylpropanoid‐mediated defense in *D. officinale*, providing a high‐resolution cellular framework for understanding plant immunity and offering potential targets for improving disease resistance in this economically valuable species.

## Significance Statement

The cellular heterogeneity of immune responses in the medicinal orchid *Dendrobium officinale* has remained largely unexplored. This single‐cell transcriptomic atlas reveals a *WRKY33*‐*HSP81* regulatory module that orchestrates phenylpropanoid‐mediated defense against fungal infection through coordinated lignin biosynthesis and transcriptional reprogramming, providing a high‐resolution framework for understanding plant immunity and offering potential targets for breeding disease‐resistant varieties of this economically valuable species.

## INTRODUCTION

*Dendrobium officinale* Kimura et Migo belongs to *Dendrobium* Orchidaceae, is a rare and endangered plant in China, known as the ‘top of the nine great medicinal herbs of China’ (Li et al., 2020; Niu et al., 2021; Zhu et al., 2018). This species not only possesses exceptional ornamental value but also boasts remarkable medicinal properties (Chen et al., 2021; Liu et al., 2021). As a prestigious traditional Chinese herbal remedy with significant nourishing benefits, the wild resources of *D. officinale* are facing depletion (Guo, 1992). Consequently, artificial propagation and intensive cultivation have become crucial measures to meet the growing demand. However, this process is vulnerable to various diseases caused by bacteria, fungi, viruses, and insects, with southern blight being a major threat (Chen et al., 2024; Li et al., 2021). Southern blight, also known as white silk disease or sclerotial disease, significantly impacts the health of plants like *D. officinale* (Chen et al., 2019; Ying et al., 2020). Current research indicates that the primary pathogens responsible for southern blight in *D. officinale* are *Sclerotium rolfsii* Sacc. and *Sclerotium delphinii* Welch, both of which belong to the phylum Basidiomycota and the subphylum Agaricomycotina (Chen et al., 2024; Cheng et al., 2022; Wu et al., 2023). *Sclerotium rolfsii* is a necrotrophic fungus with a broad host range, favoring warm and humid habitats, predominantly found in tropical and subtropical grasslands and rainforests. Under high‐temperature and humid conditions, this fungus exhibits strong infectivity, posing a severe threat to various plants, including crops and ornamental flowers (Cui et al., 2024).

Lignin, a major component of plant cell walls, is also significant in the mechanisms of plant defense against pathogens (Martim et al., 2024; Xiao et al., 2024; Zhang et al., 2024). The biosynthesis of lignin is crucial for plant resistance; following pathogen infection, the degree of lignification in cell walls increases, thereby enhancing mechanical strength and resistance to cell wall‐degrading enzymes released by pathogens (Hernández‐Blanco et al., 2007; Hückelhoven, 2007). While transcription factors (TFs) are important participants in the signaling pathways of plant disease resistance, lignin serves as a critical substance in plant defense responses. TFs play a vital role in plants' responses to biotic stressors such as insect attacks and pathogen infections (Reboledo et al., 2022). In response to these adversities, many families of TFs have been reported to show differential expression in plants facing bacterial, fungal, and viral infections (Amoroso et al., 2023; Mierziak & Wojtasik, 2024). For instance, TFs such as *HSP90* (Ko et al., 2023), *MYB* (Ahmad et al., 2024; Yu et al., 2023; Zhang, Jiang, et al., 2019; Zhang, Zhang, et al., 2019), *WRKY* (Im et al., 2022; Javed & Gao, 2023; Javed et al., 2022), *bHLH* (Huang et al., 2024; Yan et al., 2024), *NBS* (Gu et al., 2020; Si et al., 2022), *VQ* (Dong et al., 2024; Duan et al., 2024; Tian et al., 2023), *BES1* (Cao et al., 2024; Sun et al., 2022), and *bZIP* (Tian et al., 2024; Wang, Chen, et al., 2024; Wang, Luo, et al., 2024; Yin et al., 2024) have been identified as key players in responding to pathogen attacks. However, the exact role of lignin in the molecular mechanisms underlying *D. officinale*'s resistance to *S. rolfsii* remains unclear. It is currently uncertain which TFs are involved in regulating the expression of key enzyme genes in the lignin synthesis pathway, thus affecting the plant's disease resistance response. Moreover, further research is necessary to determine whether TFs enhance resistance to pathogens by regulating the expression of one or more key enzyme genes involved in lignin synthesis.

Among these, *WRKY* TFs are well‐established as key regulators of plant immunity, functioning through direct binding to W‐box elements (TTGACC/TTGACT) in the promoters of defense‐related genes (Im et al., 2022; Javed & Gao, 2023; Javed et al., 2022). *WRKY33*, in particular, has been extensively characterized in *Arabidopsis thaliana* as a master regulator of camalexin biosynthesis and resistance to necrotrophic pathogens (Birkenbihl et al., 2012). However, the functional roles of *WRKY33* in non‐model medicinal plants such as *D. officinale* remain largely unexplored. Heat shock protein 90 (*HSP90*) family members are molecular chaperones traditionally associated with protein folding, stability, and stress responses (Ko et al., 2023). Emerging evidence has implicated *HSP90* in plant immunity through its interactions with diverse client proteins, including nucleotide‐binding site‐leucine‐rich repeat (NBS‐LRR) resistance proteins and TFs (Klink et al., 2007). Nevertheless, the nuclear functions of *HSP90* family proteins in directly modulating TF activity and defense gene expression remain poorly understood.

Cells play a crucial biological role in plant growth and development, as well as in responding to environmental stresses. Single‐cell transcriptome sequencing (scRNA‐seq) technology enables parallel transcriptomic analysis of individual cells, allowing for a deeper exploration of the heterogeneity between different cell types in complex tissues and the identification of previously unknown cell types (Mazutis et al., 2013; Rich‐Griffin et al., 2020). scRNA‐seq technology has been widely applied to understand the interactions between plants and microorganisms, revealing numerous key molecular events (Cao et al., 2023; Liang et al., 2023; Serrano et al., 2024; Tang et al., 2023). Advances in cell‐type‐specific transcriptomics have uncovered critical processes in plant cells involved in stress adaptation, providing a unique opportunity to understand the heterogeneity of gene expression changes among different cell populations (Dinneny et al., 2008). Performing single‐cell transcriptomic analysis on plant tissues under pathogen invasion will facilitate the identification of the major cell populations involved in immune resistance pathways (Bai et al., 2022; Jean‐Baptiste et al., 2019).

In this study, we constructed a comprehensive single‐cell transcriptomic atlas of *D. officinale* stem bases in response to *S. rolfsii* infection, identifying six major cell types and characterizing their dynamic transcriptional changes during early and late infection stages. We discovered that *WRKY33* and *HSP81* (*HSP90* family) are key regulators with opposing functions in pathogen defense, mediated through differential modulation of lignin and flavonoid biosynthesis genes. We further demonstrated that *WRKY33* directly activates lignin biosynthetic genes *C4H* and *HCT* by binding to W‐box motifs, and that *WRKY33* physically interacts with *HSP81* in the nucleus, forming a multilayered regulatory network that integrates biotic and abiotic stress signals. Collectively, our findings establish a *WRKY33*‐*HSP81* regulatory module that orchestrates phenylpropanoid‐mediated defense against *S. rolfsii* in *D. officinale*, providing novel insights into plant stress responses and potential targets for genetic improvement of disease resistance.

## RESULTS

### Construction of transcriptome map of stem base cells of *D. officinale*

The stem base of annual *D. officinale* tissue culture seedlings was inoculated with 5 mm diameter *S. rolfsii* AGAR and the infection progression was observed. Based on observational analysis, stem base tissue collection was selected at 12 and 24 hpi after inoculation because they represent the early and late stages of infection, respectively. Protoplasts were isolated from stem base tissues of *D. officinale* in both control and experimental groups, and single‐cell RNA sequencing was performed using 10× genomics workflow (Figure S1). At least 20 000 single cells were isolated on a large scale, bound to gel beads with cell tag sequences and encased in droplets. After applying quality control measures to remove low‐quality cells, a single‐cell transcriptome of 9136 cells was obtained from the simulated (0 hpi) sample. It contained 323 564 504 reads (Figure 1a; Table S1). The sequenced reads were compared with the *D. officinale* genome, of which 88.80% were located in the genome. An average of 2160 genes and 5062 unique molecular identifiers (UMIs) were detected per cell, which are individual tags attached to each complementary DNA (cDNA) molecule to track their original origin and avoid PCR duplication (Figure 1a; Table S1). 6355 and 8378 single cells were detected in the treated samples at 12 and 24 hpi, respectively (Figure 1a; Table S1). The 12 hpi sample detected an average of 1672 genes and 4586 UMIs per cell, and the 24 hpi sample detected an average of 1573 genes and 3442 UMIs per cell, respectively (Figure 1a; Table S1). In general, the quality of scRNA‐seq data obtained from stem base of *D. officinale* was high.

**Figure 1:**
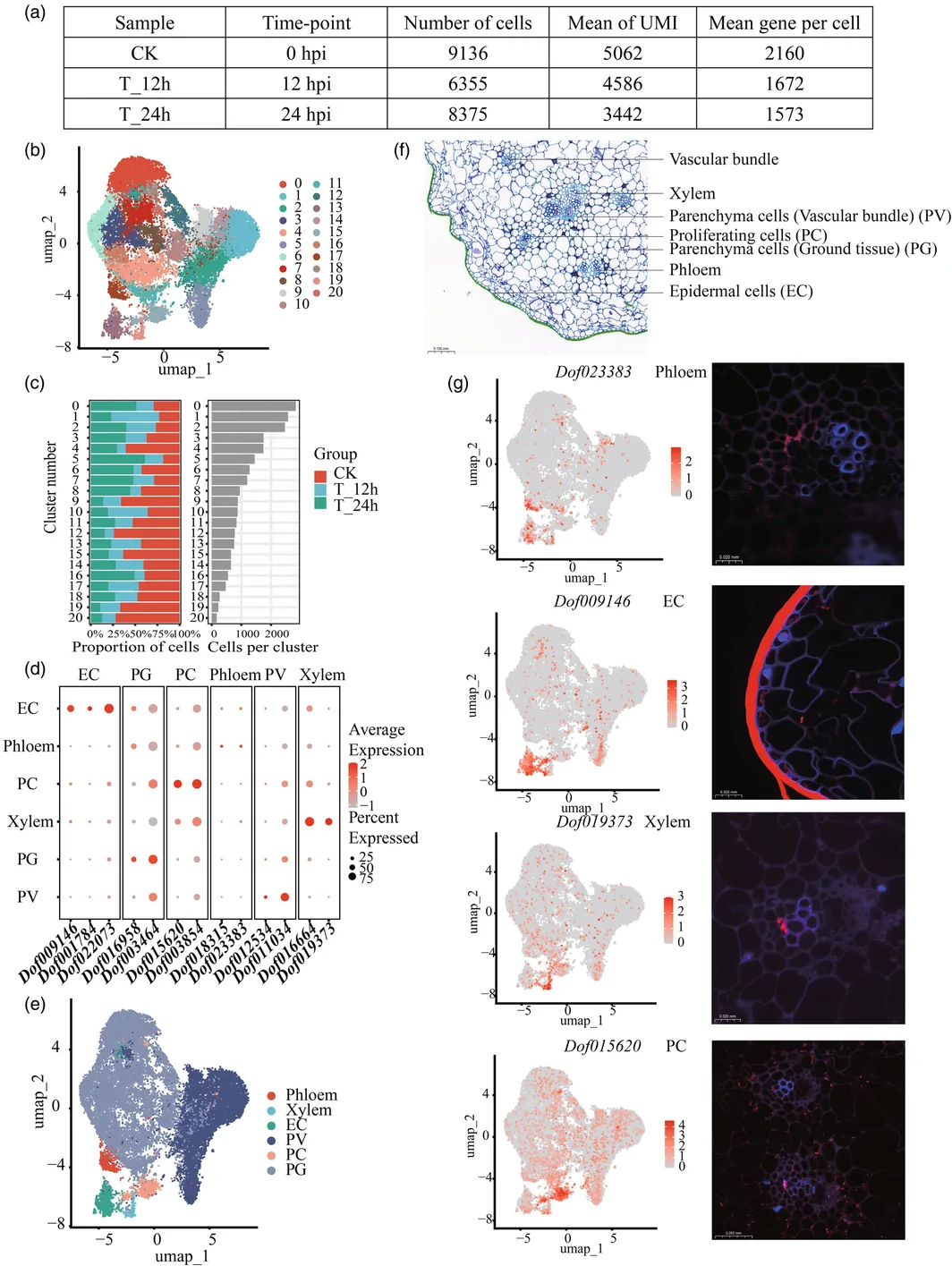
Cell heterogeneity cluster analysis of single‐cell transcriptome at stem base of *Dendrobium officinale*. (a) A summary of processed scRNA‐seq data using stem base tissue of *D. officinale* at 12 and 24 hpi after blank control or *Sclerotium rolfsii* inoculation. A unique molecular identifier (UMI) for each cell detected on the transcript. (b) Transcriptomic changes after *S. rolfsii* infection captured by scRNA‐seq. Each cell is represented by a point on a graph visualized by uniform manifold approximation and projection (UMAP). Based on transcription features, the cells were divided into 21 clusters using graph‐based unsupervised clustering. (c) Proportions of cells from CK, 12 hpi, or 24 hpi samples in each cluster and the number of cells in each cluster. The proportions were normalized against the total number of cells in each sample. (d) Expression patterns of cell‐type marker genes for each cell cluster. Dot diameter indicates the percentage of cluster cells that expressed the gene. Color indicates the average expression across cells in that cluster. (e) Visualization of six broad populations and their spatial distribution in stem base of *D. officinale* using UMAP. (f) Microscopic structure and cell type annotation of cross‐sections of *D. officinale* stems. (g) UMAP visualization and fluorescence *in situ* hybridization (FISH) analysis of four cluster‐specific marker genes in *D. officinale* stems.

Principal component analysis (PCA) reduction and cluster analysis using the scRNA‐seq dataset generated a transcriptome map containing 21 cell clusters (Figure 1b; Table S2). In order to more clearly clarify the similarities and differences between cell clusters, the unified manifold approximation and projection (UMAP) was used to visualize the cell clusters. The number of cells ranged from 2834 (cluster 0) to 171 (cluster 20) (Figure 1c; Table S2). Since there were no previous reports on the cellular heterogeneity of *D. officinale*, we used homologous genes of cluster‐specific genes from rice (*Oryza sativa* L.) and *A. thaliana* (L.) Heynh. to annotate these clusters (Figure 1d; Table S3). To achieve more accurate annotation of the corresponding cell clusters, we utilized known single‐cell markers in rice available on scPlantDB (https://biobigdata.nju.edu.cn/scplantdb/marker) to identify homologous genes in *D. officinale* (Table S3). Based on these markers, a total of six cell types were identified: epidermal cells (EC; cluster 13), parenchyma cells (Ground tissue) (PG; cluster 0, 3, 4, 6, 7, 8, 10, 11, 12, 16, 18, and 20), proliferating cells (PC; cluster 15), xylem cells (Xylem; cluster 19), phloem cells (Phloem; cluster 17), and parenchyma cells (Vascular bundle) (PV; cluster 1, 2, 5, 9, and 14) (Figure 1e,f; Figure S2; Table S4). The stem base cell map of *D. officinale* helps to further characterize the heterogeneity of these specific cell types. The fluorescence *in situ* hybridization (FISH) results were consistent with the identified cell types (Figure 1g). Accordingly, *Dof023383*, *Dof009146*, *Dof019373*, and *Dof015620* were confirmed as marker genes for phloem cells, ECs, xylem cells, and PC, respectively, in the stems of *D. officinale* (Figure 1g).

### Single‐cell heterogeneity analysis of *D. officinale* stem base

Revealing how different cell types respond to biological stress is crucial. The single‐cell expression profile of *D. officinale* stem base infected with *S. rolfsii* was characterized. The relationship between different expression profiles was explored by comparing the response of different cell types to infection. The analysis of the proportion of different cell types in CK, 12 hpi, and 24 hpi samples showed that the stem base of *D. officinale* had an obvious response to the stimulation of *S. rolfsii*. Different cell clusters showed different characteristics in the course of pathogen infection. The expression patterns of xylem cells and PC gradually decrease. Notably, epidermal and phloem cells exhibit a significant reduction in expression at 24 h, distinguishing them from other cell types (Figure 2a,b; Table S5). In order to understand the gene expression pattern of each cell type during the infective process of *D. officinale* stem base, the differentially expressed genes (DEGs) of each cell type were specifically identified at different infection time points. To determine transcriptional heterogeneity across cell types, differences in the number of DEGs between 12 and 24 hpi specific cell types were identified for six cell types. The number of DEGs in xylem tissue cells was significantly higher than that in the other five cell types (Figure 2c; Table S6). Although there is some duplication of DEGs between different cell types, most DEGs occur mainly in a single cell type. The amount of DEGs in different cell types was different during the course of pathogen infection (Figure 2d; Table S6).

**Figure 2:**
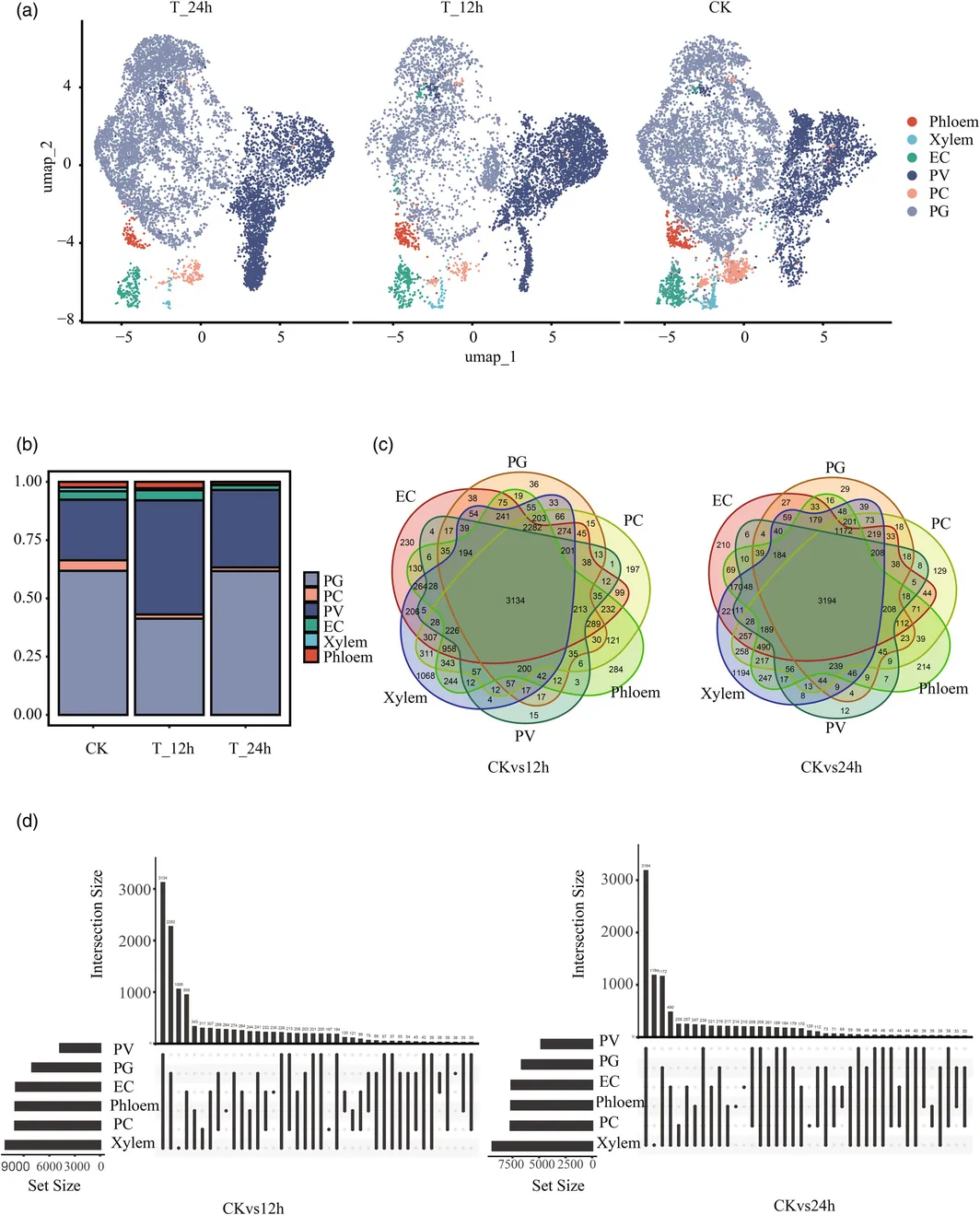
Single‐cell transcriptome analysis of infected and control samples. (a) Uniform manifold approximation and projection (UMAP) plot of separated CK, 12 hpi, and 24 hpi single cells. (b) Bar plot showing the relative proportion of each cell type in CK, 12 hpi, and 24 hpi samples. (c) The proportion of differentially expressed genes (DEGs) in different cell types at 12 and 24 hpi with *Dendrobium officinale*. (d) UpSet plot of DEGs at 12 and 24 hpi with *D. officinale*. The intersection sizes between several cell types in response to *D. officinale* are displayed on the UpSet plot. The height of the bar under each column reflects the number (intersection size) of DEGs shared by the cell types filled in the matrix.

### Expression patterns of different cell lines responding to pathogen infection

In order to further understand the expression patterns of different cell types of *D. officinale* stem base genes in response to the stimulation of *S. rolfsii*, the GO and KEGG pathways of these DEGs at two different infection time points in six different types were analyzed. At 12 and 24 hpi, immune‐related GO items were significantly enriched in six cell types. At 12 hpi, ECs, phloem cells, and parenchyma cells (Ground tissue) were enriched and significantly upregulated in the types of defense response to fungus (GO:0050832) (Figure 3a; Table S7). ECs, phloem cells, xylem cells, PC, and parenchyma cells (Ground tissue) were enriched and significantly upregulated in the glycolytic process types (GO:0006096) (Figure 3a; Table S7). ECs and parenchyma cells (Ground tissue) were enriched and significantly upregulated in the regulatory types of regulation of defense response to fungus (GO:1900150) (Figure 3a; Table S7). These results indicated that ECs, phloem cells, and parenchyma cells (Ground tissue) were most abundant at 12 hpi and were involved in the physiological process of *D. officinale*'s defense against pathogenic bacteria. At 24 hpi, the six cell types were enriched and significantly upregulated in the defense response to fungus type (GO:0050832) and the defense response to fungus (GO:1900150), and the PC were significantly enriched in the response to hydrogen peroxide (GO:0042542) (Figure 3c; Table S7). Interestingly, at 12 and 24 hpi, six cell types were enriched and significantly downregulated in protein folding (GO:0006457) and cellular response to oxidative stress (GO:0034599) (Figure 3b,d; Table S7). In the KEGG analysis, DGEs involved in the ribosome pathway (dct03010) were significantly downregulated across different cell subpopulations at both 12 and 24 hpi (Table S8). At 12 hpi, DGEs associated with the mRNA surveillance pathway (dct03015) and alpha‐linolenic acid metabolism (dct00592) were significantly upregulated in most cell subpopulations (Table S8). By 24 hpi, DGEs related to plant–pathogen interaction (dct04626) showed a significantly upregulated trend across different cell subpopulations (Table S8).

**Figure 3:**
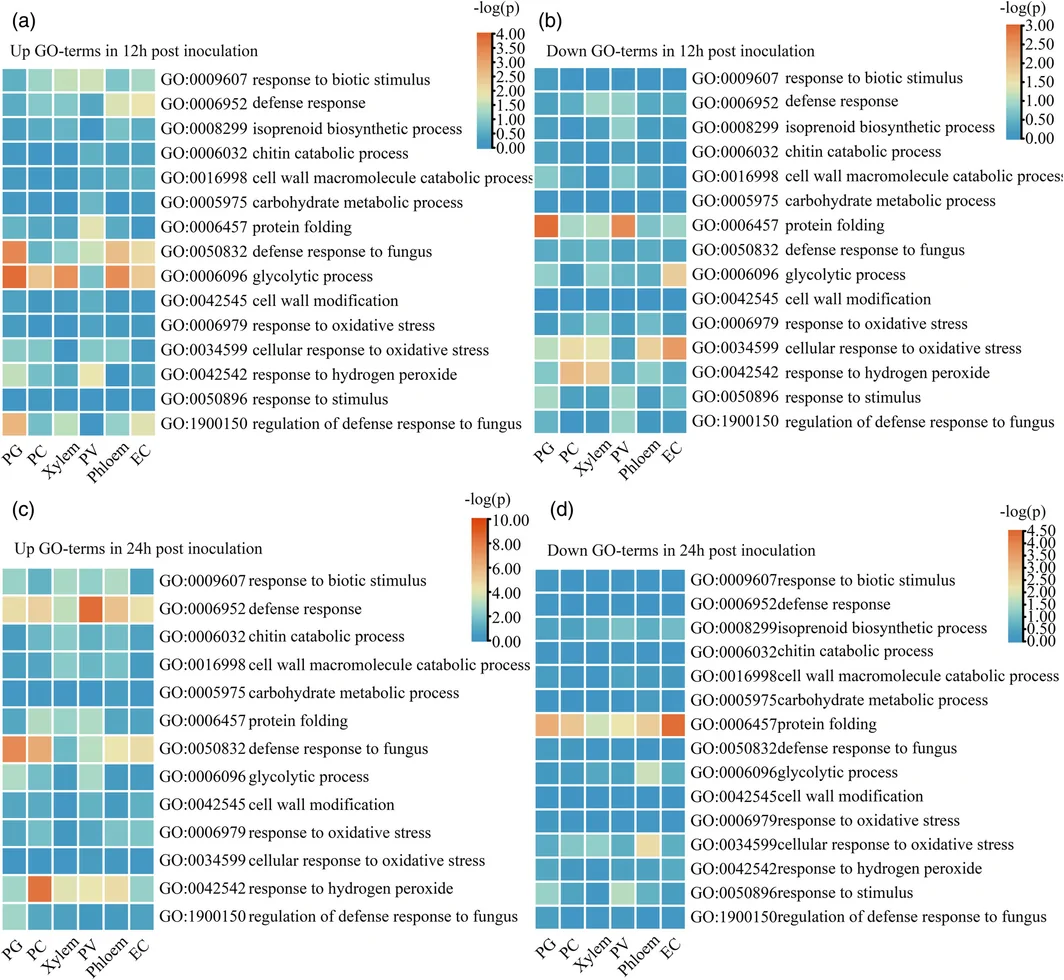
The gene ontology (GO) terms of *Dendrobium officinale* in response to *Sclerotium rolfsii* infection. (a) GO terms enriched in upregulated differentially expressed genes (DEGs) at 12 hpi in multiple cell types (*P* ≤ 0.05). (b) GO terms enriched in downregulated DEGs at 12 hpi in multiple cell types (*P* ≤ 0.05). (c) GO terms enriched in upregulated DEGs at 24 hpi in multiple cell types (*P* ≤ 0.05). (d) GO terms enriched in downregulated DEGs at 24 hpi in multiple cell types (*P* ≤ 0.05).

### Cell trajectory analysis of five cell types at different stages of infection with *S. rolfsii* from *D. officinale*

In order to evaluate the expression profile of *D. officinale* stem base in the early and late stages of infection with *S. rolfsii*, pseudotemporal analysis was performed for five cell types (xylem cells, phloem cells, PC, ECs, and parenchyma cells (Vascular bundle)) after infection. The five cell types in the three samples were projected onto the pseudo‐time trajectory, and according to the numerical values, the developmental starting point was determined, located at the far left of the figure (Figure 4a,c). The pseudo‐time trajectory included three trajectory states (Figure 4b). The pseudo‐time trajectory of cells in the three stages was presented separately, and the figure showed that, with the increase of treatment time, cells gradually moved from state 3 and 2 to state 1 (Figure 4c,d), which can reflect the process of infection of the stem base of *D. officinale* with *S. rolfsii*. Monocle 2 was used to calculate the gene expression profiles of cells in different states with pseudotime. The DEGs can be divided into four clusters, reflecting the variation of DEGs with the change of pseudo‐time (Figure 4e). The biological pathways related to biotic stress, such as defense responses, jasmonic acid‐mediated signaling pathway regulation, abscisic acid‐activated signaling pathways, and signal receptor activity, are concentrated in clusters 2 and 4 (Figure 4e). These pathways exhibit similar expression patterns, showing an upward trend (Figure 4e).

**Figure 4:**
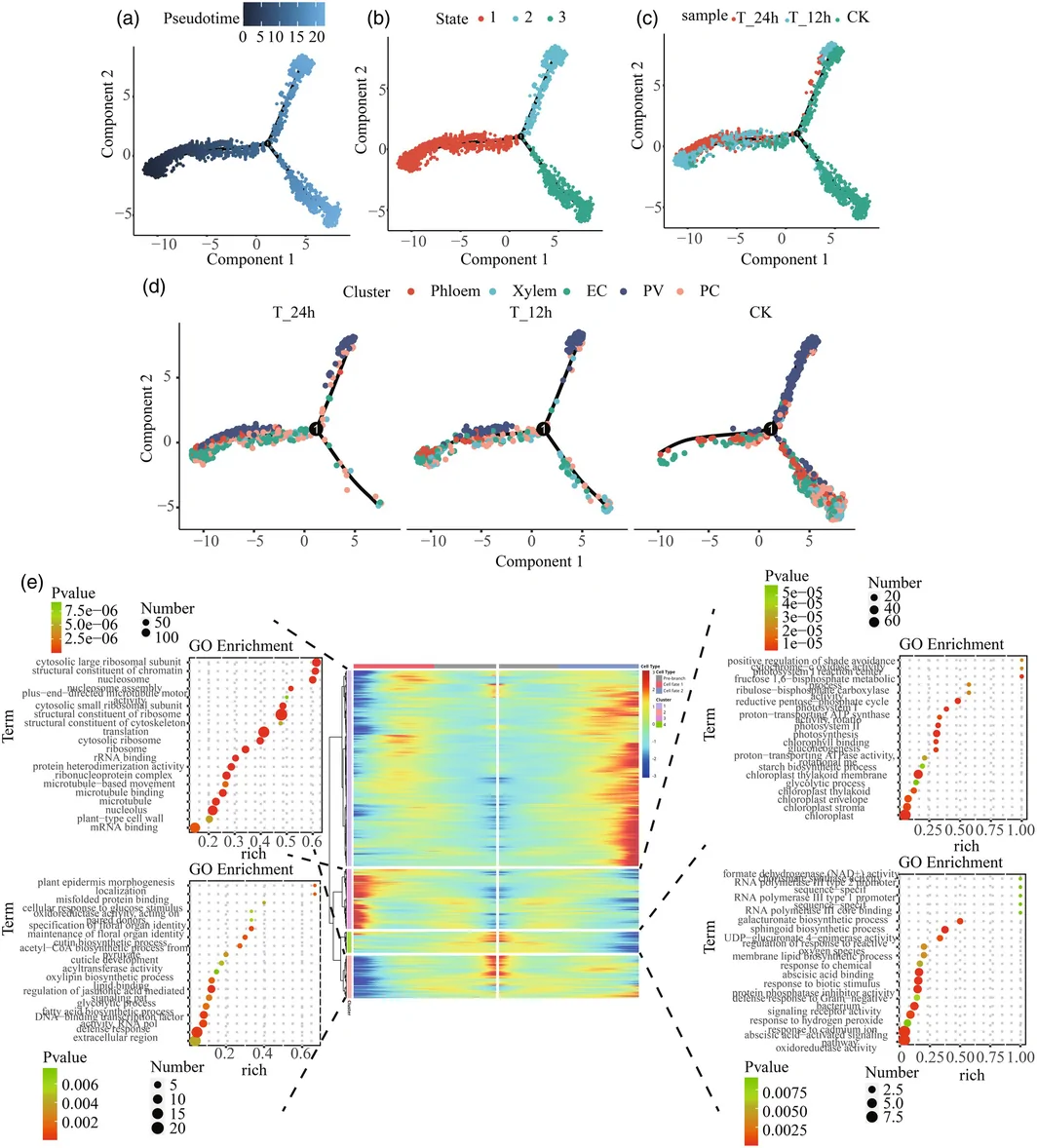
Differentiation trajectory and the fate of five cell types of 12 and 24 hpi samples using pseudotime analysis. (a) Pseudotime trajectory. Each dot represents a single cell. Color represents the pseudotime score. (b) Pseudotime trajectory. Each dot represents a single cell. Color represents different states. (c) The distribution in the trajectories of CK, T_12h, and T_24h samples. (d) The distribution of five cell types in the trajectories of CK, T_12h, and T_24h samples. (e) Pseudotime heatmap of gene ontology (GO) analysis of differentially expressed genes. Color bar indicates the relative gene expression level.

### WGCNA of *D. officinale* genes in *S. rolfsii* treatment

In this study, utilizing weighted gene co‐expression network analysis (WGCNA), four modules were identified from the DEGs in transcriptomic data from three groups of *D. officinale*. These modules comprised a total of 18 000 genes, with the largest being the Turquoise module containing 7871 genes, followed by the Blue module with 6643 genes, the Brown module with 3204 genes, and the smallest, the Purple module, with 4882 genes (Figure S4A; Table S9). PCA and hierarchical clustering dendrograms demonstrated that all nine samples clustered appropriately, with the three replicates from each group clustering together, and no outliers were detected (Figure S4B,C). As illustrated in Figure S4D, no abnormal genes were identified within the gene clusters.

### Expression patterns of transcription factors related to biological stress

During infection, TFs exhibit distinct expression patterns across various cell types, revealing complex regulatory mechanisms. Analysis of Bulk RNA‐seq data identified significant upregulation of multiple TF families, including *NBS*, *HSP90*, *bZIP*, *WRKY*, *VQ*, and *NAC*, in the basal stem region of *D. officinale* at 12 and 24 hpi (Figure 5a,b). Among these, *HSP90* (*HSP81*; *Dof002881*) was notably predominant in xylem cells, showing a steady increase in expression over time, suggesting its role in mediating stress responses and maintaining cellular homeostasis during pathogen invasion (Figure 5c; Figure S3). Similarly, *WRKY* (*WRKY33*; *Dof014177*) showed a marked increase in expression at 12 and 24 hpi compared with the control group, indicating its active involvement in orchestrating immune responses during early infection stages (Figure 5c; Figure S3). The *VQ* family member (*Dof018941*) also demonstrated tissue‐specific upregulation, particularly in xylem cells, hinting at a specialized function in regulating signaling pathways under biotic stress conditions (Figure 5c; Figure S3). Furthermore, *MYB* (*MYB78*; *Dof013141*) exhibited a more widespread expression pattern, being upregulated across multiple cell types, indicating its diverse regulatory roles in modulating cell‐specific responses to environmental stimuli (Figure 5c; Figure S3). Interestingly, the *bHLH* family member (*bHLH35*; *Dof006253*) displays a more dynamic expression pattern across various cell types. Its expression initially decreases and then increases during the treatment, suggesting a complex regulatory mechanism that might involve feedback loops or phase‐specific responses to infection (Figure 5c; Figure S3). These observations highlight the intricate interplay of TFs in modulating *D. officinale*'s defense mechanisms, with each family potentially contributing uniquely to the plant's resilience against infection.

**Figure 5:**
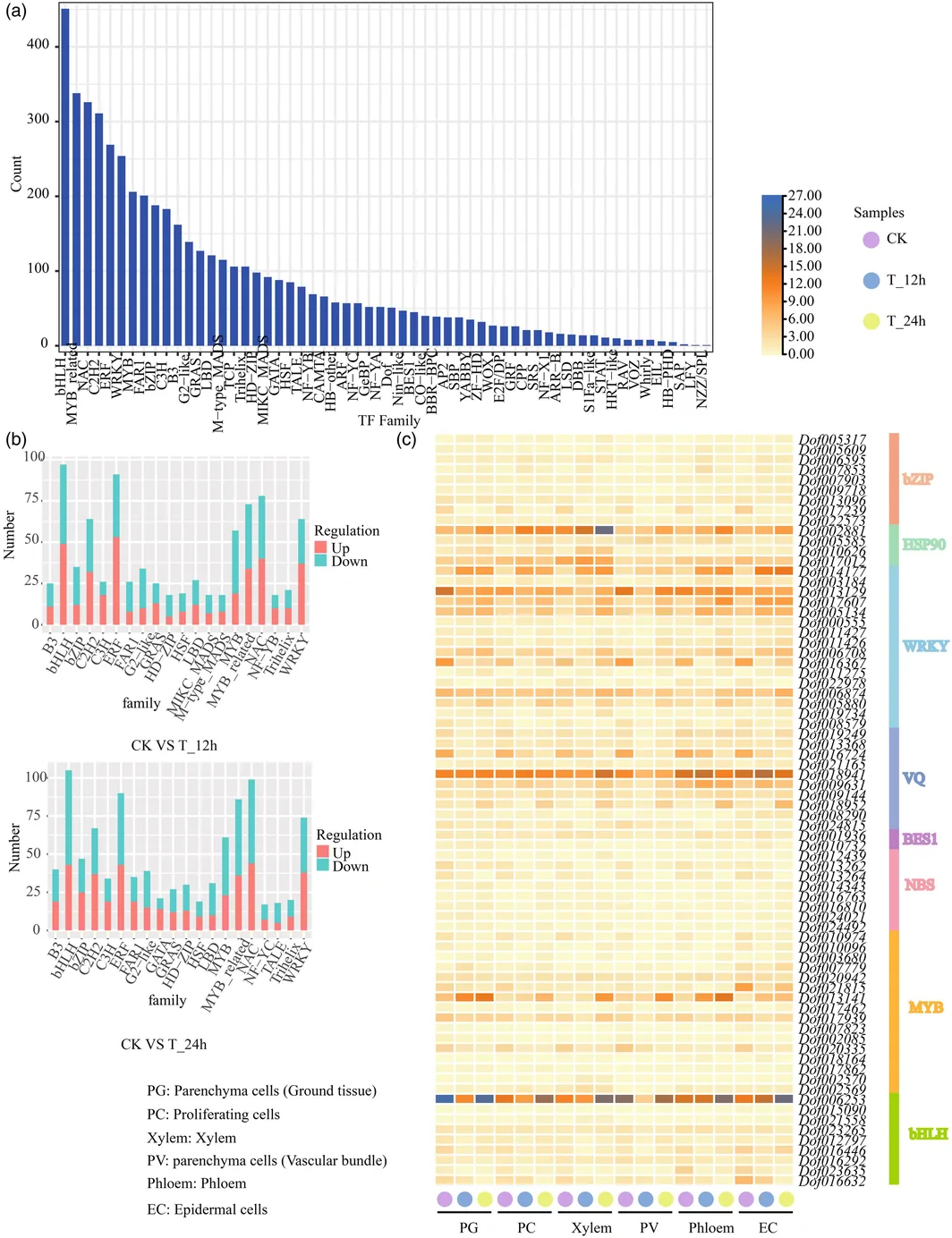
Statistics and expression analysis of different transcription factor families. (a) Transcription factor family statistics. The *x*‐axis represents different transcription factor families, while the *y*‐axis represents the number of genes associated with each transcription factor family. (b) Differential transcription factor bar chart. The *x*‐axis represents different transcription factor families, while the *y*‐axis represents the number of genes associated with each transcription factor family. (c) Heatmap of expression levels of members from different transcription factor families across various cell types.

### Regulation of the phenylpropanoid biosynthesis pathway

Lignin is a fundamental component of plant cell walls, playing a pivotal role in maintaining their structural integrity and rigidity. Additionally, the composition of plant cell walls and their dynamic interactions with pathogens are crucial for understanding plant disease resistance. Research on plant resistance mechanisms often focuses on these walls, as their molecular composition and adaptive responses are key to combating infections. The lignin metabolic pathway, a critical branch of plant defense, is intricately regulated by upstream signals that activate a cascade of reactions culminating in lignin synthesis. This pathway is part of the larger phenylpropanoid biosynthesis pathway, which branches into two distinct yet interrelated directions: the flavonoid biosynthesis branch and the lignin biosynthesis branch (Figure 6a). The flavonoid pathway is complex, consisting of six major branches that lead to the synthesis of various bioactive compounds, while the lignin pathway is more streamlined, comprising four primary branches dedicated to lignin formation (Figure 6a). Through detailed genomic research, we identified six key DEGs associated with the flavonoid biosynthesis process, including chalcone synthase (*CHS*), chalcone isomerase (*CHI*), naringenin 3‐dioxygenase (*F3H*), bifunctional dihydroflavonol 4‐reductase (*DFR*), and anthocyanidin synthase (*ANS*) (Figure 6b). Notably, *CHI* (*Dof014385*) exhibits significant differential expression, characterized by upregulation at 12 h and downregulation at 24 h, indicating a distinct pattern of initial upregulation followed by downregulation. This expression trend is particularly evident in ECs, PC, xylem cells, and phloem cells (Figure 6b). Additionally, we identified 30 crucial DEGs involved in the lignin biosynthesis process, such as 4‐coumarate‐CoA ligase (*4CL*), caffeic acid 3‐O‐methyltransferase (*COMT*), cinnamoyl‐CoA reductase (*CCR*), cinnamyl‐alcohol dehydrogenase (*CAD*), *trans*‐cinnamate 4‐monooxygenase (*C4H*), shikimate O‐hydroxycinnamoyltransferase (*HCT*), ferulate‐5‐hydroxylase (*F5H*), and *peroxidase*, all of which play specific roles in lignin production (Figure 6d). During the treatment process, the expression levels of *4CL* (*Dof005163*), *CCR* (*Dof005734*), *C4H* (*Dof003361*), *HCT* (*Dof008135*), *F5H* (*Dof003845*), and *peroxidase* (*Dof014188*; *Dof022187*) exhibited an upward trend. Notably, *C4H* (*Dof003361*) showed high expression specifically in the phloem and ECs at 12 hpi (Figure 6d). Further investigation using gene ontology (GO) enrichment analysis revealed that these DEGs involved in lignin biosynthesis fall into five major functional categories (Figure 6c). These categories include the phenylpropanoid metabolic process, directly tied to lignin production, the plant's response to antibiotics and external stimuli, transferase activity (particularly those transferring acyl groups other than amino‐acyl groups), and processes related to the plant‐type cell wall (Figure 6c). Overall, the lignin metabolic pathway is a vital component of plant defense strategies, illustrating the complex yet elegant nature of plant biochemical networks in protecting against pathogen invasion.

**Figure 6:**
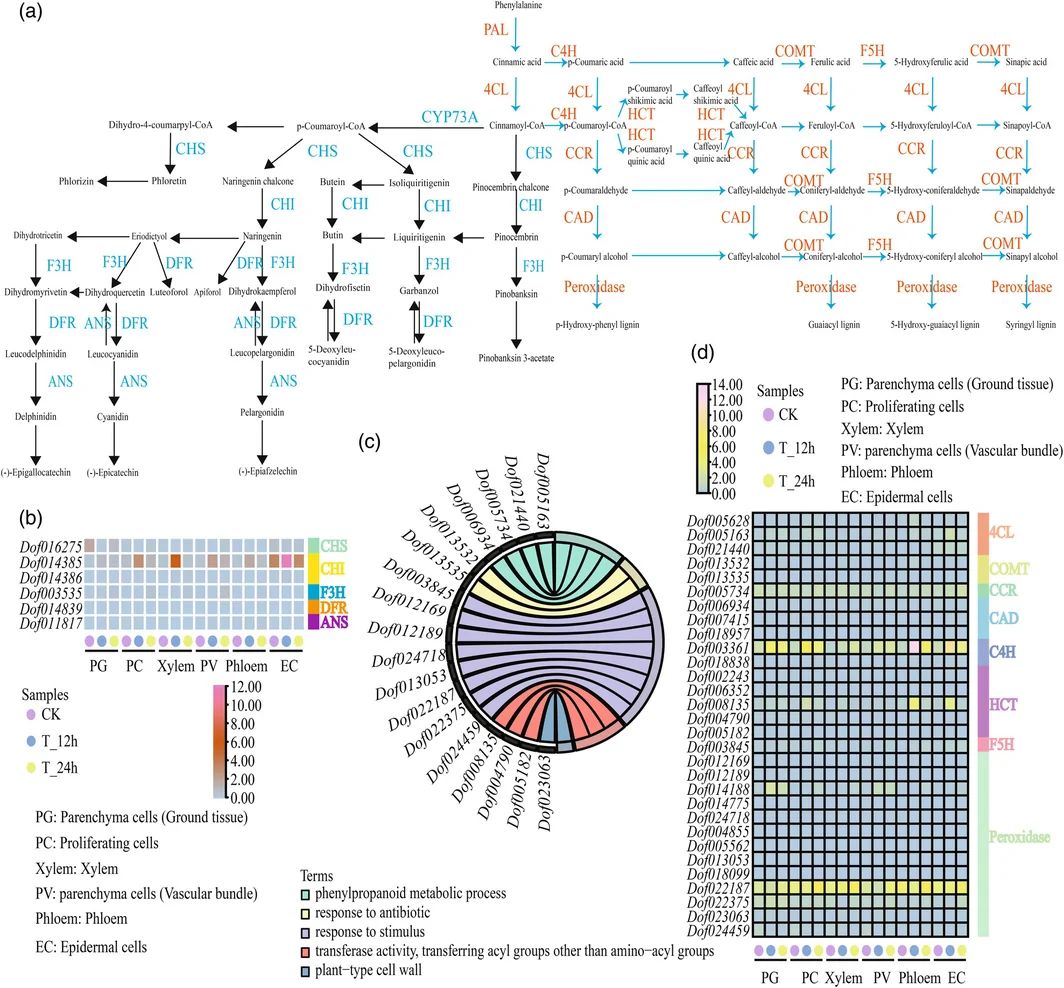
Identification and expression analysis of key enzyme genes in the phenylpropanoid biosynthesis pathway. (a) The two downstream branches of lignin and flavonoid biosynthesis in the phenylpropanoid biosynthesis pathway. (b) Heatmap of key enzyme gene expression in the flavonoid biosynthesis pathway across six different cell types. (c) Gene ontology (GO) enrichment analysis of key enzyme genes in the lignin biosynthesis pathway. (d) Heatmap of key enzyme gene expression in the lignin biosynthesis pathway across six different cell types.

### *WRKY33* enhances pathogen resistance while *HSP81* compromises defense via differential regulation of phenylpropanoid pathway genes

To assess the functional impact of *HSP81* and *WRKY33* on pathogen resistance, overexpression constructs of each gene were individually introduced into *D. officinale* leaves. Twenty‐four hours post‐transformation, a 5 mm mycelial plug of *S. rolfsii* was inoculated onto the leaves. Notably, leaves overexpressing *WRKY33* exhibited a significantly smaller lesion area compared with the control, indicating enhanced resistance (Figure 7a,b). In contrast, *HSP81*‐overexpressing leaves displayed a markedly larger lesion area, suggesting compromised defense (Figure 7a,b). To elucidate the molecular mechanisms underlying these contrasting phenotypes, quantitative PCR (qPCR) was performed to examine the expression of key genes involved in lignin and flavonoid biosynthesis. Lignin‐related genes analyzed included *4CL* (*Dof005163*), *CCR* (*Dof005734*), *CAD* (*Dof006934*), *C4H* (*Dof003361*), *HCT* (*Dof008135*), *COMT* (*Dof013532*), *F5H* (*Dof003845*), and *peroxidase* (*Dof014188*, *Dof022187*). Flavonoid‐related genes assessed included *CHS* (*Dof016275*), *CHI* (*Dof014386*), *F3H* (*Dof003535*), and *DFR* (*Dof014839*). In *WRKY33*‐overexpressing leaves, lignin‐related genes including *C4H*, *HCT*, and *COMT* were significantly upregulated, whereas flavonoid biosynthesis genes such as *4CL*, *CHS*, *F3H*, and *DFR* were significantly downregulated (Figure 7c). In HSP81‐overexpressing leaves, qPCR analysis revealed significant downregulation of *4CL*, *CHI*, *CAD*, *F5H*, and *peroxidase* (*Dof022187*), while *DFR* and *HCT* were significantly upregulated; *C4H* expression showed no significant change, and the remaining genes examined were unaffected (Figure 7c). Collectively, these results demonstrate that *WRKY33* and *HSP81* play distinct and opposing roles in regulating the phenylpropanoid biosynthesis pathway, differentially modulating lignin and flavonoid production and ultimately influencing the plant's response to pathogen infection.

**Figure 7:**
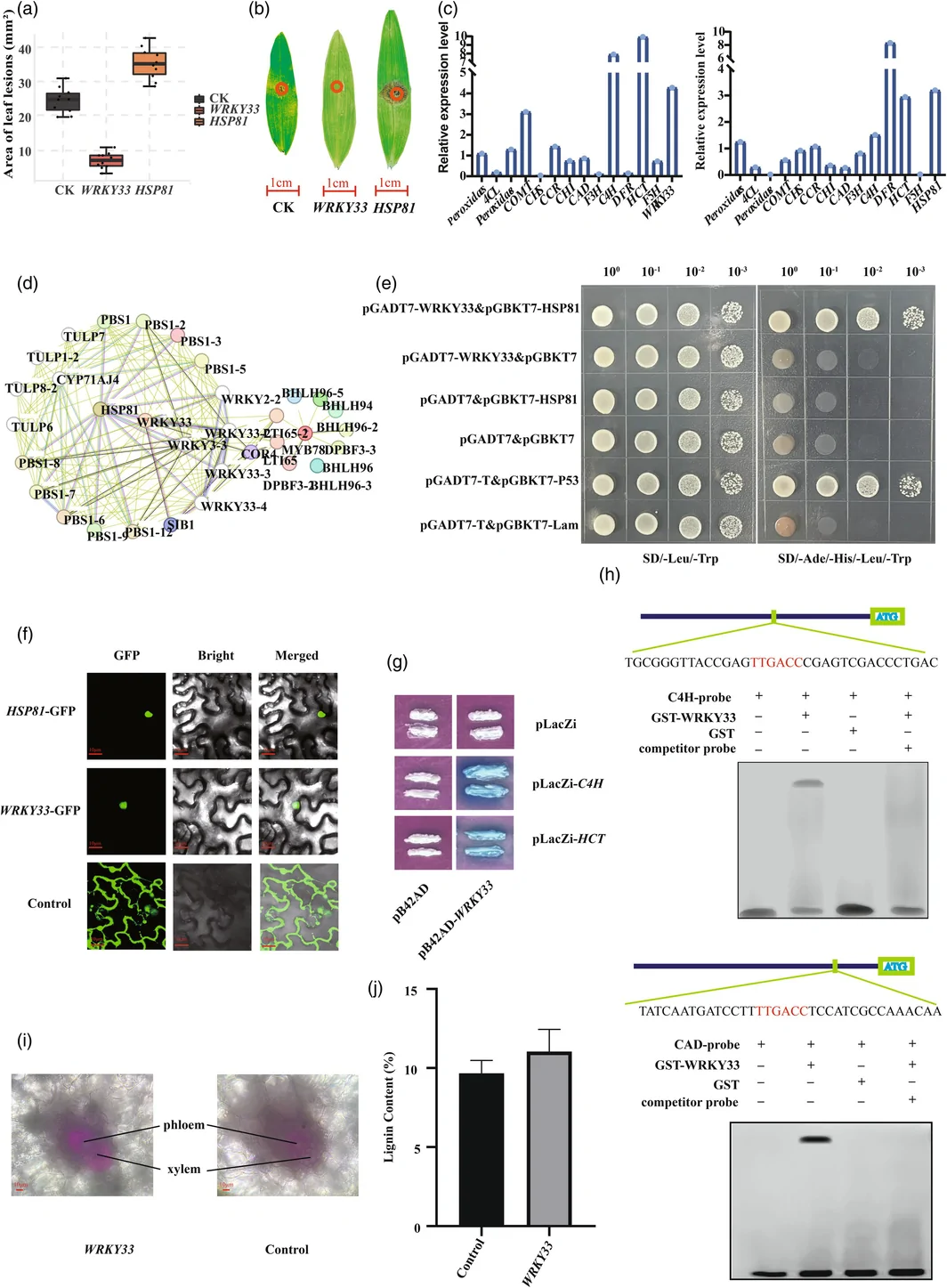
*WRKY33* positively regulates lignin biosynthesis and pathogen resistance. (a, b) The two transcription factors (*HSP81* and *WRKY33*) were overexpressed in the leaves, and disease symptoms were monitored on the third day after inoculation with *Sclerotium rolfsii*. The lesion area on each inoculated leaf was measured. (c) qPCR analysis of lignin and flavonoid biosynthesis genes in *WRKY33*‐OE and *HSP81*‐OE lines. (d) Protein–protein interaction network. (e) Yeast two‐hybrid assay confirming *HSP81*‐*WRKY33* interaction. (f) Subcellular localization of *HSP81*‐GFP and *WRKY33*‐GFP in *Nicotiana benthamiana* epidermal cells. (g) Yeast one‐hybrid assay showing *WRKY33* activation of *C4H* and *HCT* promoters. (h) Electrophoretic mobility shift assays (EMSA) demonstrating direct binding of *WRKY33* to W‐box motifs in *C4H* and *HCT* promoters. (i) Wiesner staining of stem cross‐sections showing enhanced lignin deposition in *WRKY33*‐OE lines (scale bar = 10 μm). (j) Quantitative lignin content measured by acetyl bromide method. Data represent mean ± SEM (*n* = 3; *P* < 0.01, *t*‐test).

### A multilayered protein–protein interaction network mediates coordinated stress responses in *D. officinale*

Protein–protein interaction (PPI) network analysis revealed a complex regulatory module centered on *HSP81*, *WRKY33*, and *LTI65* in *D. officinale*. Yeast two‐hybrid (Y2H) assays validated that *HSP81*, a heat shock protein functioning as a molecular chaperone involved in protein folding and stability maintenance, physically interacts with the TF *WRKY33* (Figure 7e). This interaction is proposed to enhance the transcriptional regulatory activity of WRKY33 under stress conditions. Furthermore, *WRKY33* was found to interact with *LTI65* (low‐temperature‐induced protein 65), a protein associated with cold stress responses (Figure 7d; Table S11), suggesting a coordinated role of these TFs in integrating multiple stress signaling pathways. Collectively, these findings uncover a complex and multilayered PPI network that enables plants to mount coordinated responses to diverse environmental stresses.

### Exclusive nuclear localization of *HSP81* and *WRKY33* suggests their roles in transcriptional regulation

To determine the subcellular localization of *HSP81* and *WRKY33*, a transient expression system was employed in *Nicotiana benthamiana* leaves. The coding sequences of *HSP81* and *WRKY33* were fused in‐frame with GFP at their N‐termini, placed under the control of the CaMV 35S promoter, and introduced into leaf ECs via *Agrobacterium*‐mediated transformation. Transient expression of the fusion proteins revealed that both *HSP81* and *WRKY33* are exclusively localized in the nucleus (Figure 7f). The nuclear localization of *HSP81*, a molecular chaperone, suggests its potential involvement in regulating stress‐responsive gene expression by interacting with TFs, regulatory proteins, or chromatin remodeling complexes (Figure 7f). Similarly, the nuclear presence of *WRKY33* positions it to directly regulate specific gene expression during plant development and stress adaptation (Figure 7f). Collectively, these findings indicate that both *HSP81* and *WRKY33* function within the nucleus to coordinately orchestrate transcriptional programs governing plant growth, development, and stress responses, likely through activating or repressing the transcription of target genes.

### *WRKY33* directly binds to and activates transcription of C4H and HCT promoters

To investigate whether *WRKY33* transcriptionally activates the key phenylpropanoid pathway genes *C4H* and *HCT*, a yeast one‐hybrid (Y1H) assay was performed. Putative W‐box binding sites (TTGACC/TTGACT) were identified within the 2000 bp promoter regions upstream of *C4H* and *HCT*. The Y1H results showed that *WRKY33* induced a positive color reaction in the presence of both pro*C4H* and pro*HCT*, indicating transcriptional activation of these promoters (Figure 7g). No color reaction was observed in the blank control group, nor in transformants harboring pB42AD‐*WRKY33*, pro*C4H*‐LacZi, or pro*HCT*‐LacZi alone (Figure 7g). Consistent with these findings, electrophoretic mobility shift assays (EMSA) were conducted using the *WRKY33*‐GST fusion protein and promoter fragments of *C4H* and *HCT* containing the W‐box motifs. The results demonstrated that *WRKY33*‐GST specifically bound to the W‐box sequences within both promoters, further supporting direct regulation (Figure 7h).

### WRKY33 regulates lignin synthesis

To explore the regulatory function of the *WRKY33* TF in lignin biosynthesis within the phenylpropanoid pathway, Wiesner staining was performed on cross‐sections of *D. officinale* stems from both *WRKY33*‐overexpressing lines and control plants. Compared with the control, *WRKY33*‐overexpressing stems exhibited a notably deeper red coloration, indicating enhanced lignin deposition in vascular bundles (Figure 7i). Consistent with this histological observation, quantification of lignin content using the acetyl bromide method revealed that *WRKY33*‐overexpressing stems accumulated significantly higher lignin levels (11.04%) than control stems (9.67%), confirming that *WRKY33* positively regulates lignin biosynthesis (Figure 7j).

## DISCUSSION

### Single‐cell atlas construction reveals cellular heterogeneity in the *D. officinale* stem base

In this study, we successfully isolated high‐quality protoplasts from the stem base of *D. officinale* and constructed the first single‐cell transcriptomic atlas for this medicinally important orchid species. Through unbiased clustering and dimensional reduction, we resolved 21 distinct cell clusters, which were annotated into 6 major cell types based on homology to known marker genes from *A. thaliana* and *O. sativa*, combined with detailed anatomical characterization of stem cross‐sections (Figure 1b,c,e; Table S2). This comprehensive annotation was further validated by FISH, confirming the specificity of marker genes for phloem cells (*Dof023383*), ECs (*Dof009146*), xylem cells (*Dof019373*), and PC (*Dof015620*) (Figure 1g).

The six identified cell types—EC, ground tissue parenchyma cells (PG), PC, xylem cells, phloem cells, and vascular bundle parenchyma cells (PV)—reflect the complex anatomical organization of the monocot stem. ECs, characterized by thickened and slightly lignified walls covered by a cuticle, serve as the primary physical barrier against environmental stresses (Arya et al., 2021; Bock et al., 2021). Ground tissue parenchyma cells, which constitute the bulk of the stem, are polygonal and vary in size, with non‐lignified walls, functioning primarily in storage and lateral transport (Yu et al., 2017; Yuan et al., 2019). The vascular bundles, comprising xylem, phloem, and vascular bundle parenchyma cells, are widely and complexly distributed throughout the stem, with lignified sheaths and fiber cells arranged on the outer side of the phloem or inner side of the xylem.

Notably, the generation of a comprehensive marker gene library specific to the stem base of *D. officinale* (Table S2) represents a valuable resource for future functional studies. This single‐cell atlas not only provides a foundation for investigating cell‐type‐specific biological processes but also enables precise targeting of candidate genes involved in secondary metabolism, development, and stress responses. Given the economic and medicinal importance of *D. officinale*, this resource will facilitate the discovery of genes underlying key agronomic traits and support the development of molecular breeding strategies.

### Cell‐type‐specific responses to *S. rolfsii* infection reveal a coordinated defense strategy

Plants exhibit age‐dependent resistance, with mature plants demonstrating significantly enhanced pathogen resistance compared with seedlings (Bruns et al., 2022; Millett et al., 2009). In this study, we selected 1‐year‐old tissue‐cultured seedlings to investigate the cellular and molecular responses to *S. rolfsii* infection at 12 and 24 hpi, representing early and late infection stages. Our single‐cell analysis revealed dynamic and cell‐type‐specific transcriptional reprogramming during the infection process.

The proportional changes in cell types across the three time points indicated distinct responses to pathogen challenge. Notably, the expression patterns of xylem cells and PC gradually decreased over time, while epidermal and phloem cells exhibited a significant reduction at 24 hpi, distinguishing them from other cell types (Figure 2a,b; Table S5). ECs, as the outermost cell layer, are covered by a hydrophobic cuticle that serves as the first line of defense against fungal and bacterial pathogens (Arya & Cohen, 2022; Arya et al., 2021; Bock et al., 2021). To successfully colonize, pathogens must overcome this barrier. Our findings suggest that the coordinated response of meristematic and ECs is closely associated with resistance to *S. rolfsii*. The reduction in EC abundance at 24 hpi may reflect their role as frontline defenders, with some cells undergoing programmed cell death to limit pathogen spread—a common feature of the hypersensitive response (HR) (Rymen & Sugimoto, 2012).

The vascular bundle, composed of xylem and phloem, plays essential roles in water and nutrient transport, antimicrobial substance translocation (including phenolic compounds and antimicrobial proteins), and immune signal transduction (Mazumdar et al., 2025; Tancos et al., 2018). Upon pathogen invasion, the vascular bundle rapidly transports antimicrobial compounds synthesized by other tissues (such as ECs) to infection sites, enhancing local immunity (Walerowski et al., 2018). Furthermore, immune signals activated by ECs can be transmitted through the vascular bundle to distal tissues, regulating systemic disease resistance. The observed reduction in phloem cell abundance at 24 hpi may reflect the dynamic nature of phloem‐mediated signaling and resource allocation during the later stages of infection.

The collaborative action of ECs and vascular bundles is a key mechanism for enhancing disease resistance in *D. officinale*. ECs provide physical and chemical barriers against pathogen invasion, while vascular bundles contribute through antimicrobial substance transport, immune signal regulation, and structural reinforcement. When attacked by pathogens, both ECs and vascular bundles trigger lignification, increasing lignin deposition and thereby enhancing cell wall rigidity (Wang, Chen, et al., 2024; Wang, Luo, et al., 2024). Xylem lignification not only increases structural strength but also forms a barrier at infection sites, limiting pathogen spread. This coordinated defense strategy ensures rapid and effective responses to pathogen threats, enhancing the plant's competitive survival.

### *HSP81* broadly regulates multiple transcription factors via co‐regulator competition

The exclusive nuclear localization of *HSP81* positions this molecular chaperone to potentially interact with a diverse array of client proteins extending well beyond *WRKY33*. *HSP90* family proteins are known to bind dozens to hundreds of client proteins, many of which function as TFs or signaling components (Chen et al., 2014). Consequently, overexpression of *HSP81* may titrate not only *WRKY33* but also a broad spectrum of other nuclear proteins, including transcriptional co‐activators, co‐repressors, chromatin remodelers, and components of the basal transcription machinery (Guo et al., 2008; Kindgren et al., 2011; Samaha et al., 2010).

This co‐factor sequestration model is strongly supported by the distinct and non‐reciprocal gene expression profiles observed in *HSP81*‐overexpressing plants relative to WRKY33‐overexpressing plants. If *HSP81* functioned solely as a dedicated inhibitor of *WRKY33*, the expression profile resulting from *HSP81* overexpression would be expected to roughly mirror the inverse of the *WRKY33* overexpression profile. Specifically, genes upregulated by *WRKY33* (*C4H*, *HCT*, *COMT*) should be downregulated by *HSP81*, whereas genes downregulated by *WRKY33* (*4CL*, *CHS*, *F3H*, *DFR*) should be upregulated by *HSP81*. However, this predicted inverse relationship is not observed. Notably, while *WRKY33* downregulates *DFR*, *HSP81* upregulates *DFR*; and while *WRKY33* upregulates *HCT*, *HSP81* also upregulates *HCT*—a co‐directional rather than antagonistic effect. Furthermore, HSP81 downregulates several genes (*4CL*, *CHI*, *CAD*, *F5H*, *peroxidase*) that are either unaffected or not oppositely regulated by *WRKY33*. These discrepancies cannot be reconciled with a simple *WRKY33*‐inhibition model and instead suggest that *HSP81* exerts broader, gene‐specific effects that likely involve additional TFs.

Which other TFs might be affected by *HSP81* overexpression? A growing body of evidence points to the involvement of multiple transcriptional regulators in phenylpropanoid metabolism. Zhu and Li (2021) reported a transcriptional regulatory network centered on the NAC‐*MYB* module that governs the synthesis of lignin, cellulose, and hemicellulose, thereby promoting secondary cell wall formation and accelerating plant lignification and maturation. This network includes several *MYB* TFs—such as *MYB26*, *MYB46*, *MYB63*, *MYB85*, and *MYB103*—that have been shown to directly or indirectly regulate lignin biosynthesis (Xiao et al., 2021; Zhong & Ye, 2015). *MYB* TFs are also well‐established regulators of flavonoid biosynthesis. For instance, multiple *MYB* family members can regulate the expression of *DFR*, *ANS*, and *ANR*, thereby controlling anthocyanin accumulation (Jiao et al., 2023; Zhao et al., 2023). In addition, other TF families, including *WRKY* and *bZIP*, may influence the metabolic flux balance between lignin and flavonoid branches within the phenylpropanoid pathway (Teng et al., 2021; Wang, Luo, et al., 2024; Zhang, Zhang, et al., 2019). In the present study, overexpression of the *MYB* TF *MYB78* in *D. officinale* significantly upregulated key enzyme genes in both lignin and flavonoid biosynthesis pathways, correlating with enhanced pathogen resistance.

Collectively, these findings support a model in which *WRKY33* regulates the balance between lignin and flavonoid biosynthesis in *D. officinale* by controlling core factors such as *HCT*, thereby modulating the production of bioactive compounds as well as lignin, cellulose, and hemicellulose. This regulatory circuit influences plant tenderness, growth, and development while ultimately enhancing pathogen resistance.

In conclusion, rather than functioning solely as a dedicated inhibitor of *WRKY33*, *HSP81* appears to act as a nuclear sequestration hub that modulates the activity of multiple TFs by governing access to limiting co‐regulatory resources. This perspective elevates *HSP81* from a passive molecular chaperone to an active participant in the intricate transcriptional regulatory network that governs plant defense metabolism.

## MATERIALS AND METHODS

### Plant material, growth conditions, and fungal treatment

The biological stress was induced by infecting healthy, 1‐year‐old tissue‐cultured *D. officinale* seedlings with *S. rolfsii*. The *S. rolfsii* strain was obtained from Professor's laboratory at the College of Life Sciences, Nanjing Agricultural University (Nanjing, China). The fungus was inoculated onto potato dextrose agar (PDA) and cultivated at 25°C for 3–4 days until the mycelium completely covered the plates. A 5 mm mycelial plug was then inoculated at the base of each *D. officinale* seedling. Samples from the base of the seedlings were collected at 0, 12, and 24 h post‐treatment for subsequent protoplast preparation.

### Protoplast isolation

The collected *D. officinale* stem base tissues were cut into 1–2 mm strips and placed into test tubes containing 5% cellulase R10 (10 000 U g^−1^, Yakult, Tokyo, Japan), 2% pectinase Y‐23 (1000 U g^−1^, Yakult), 5% BSA, 0.08 mM MES, and 0.8% mannitol (without Ca^2+^ and Mg^2+^). The dissociation temperature was set at 27°C, with a pH of 5.7, and the mixture was shaken at 80 × ***g*** for 3 h. The mixture was then filtered through a 40 μm cell strainer. Cell viability was assessed using the trypan blue staining method, while cell concentration was determined using a hemocytometer and optical microscope. Finally, the protoplasts were resuspended in an 8% (w/v) mannitol solution, preparing them for loading into the chromium controller of the 10× genomics platform. Approximately 2 × 10^4^ isolated single cells and enzyme gel beads were encapsulated in a single oil droplet for single‐cell RNA‐seq library construction.

### Chromium 10x Genomics library and sequencing

Cells were loaded onto the 10x Chromium Single Cell Platform (10x Genomics, Pleasanton, CA, USA) at a concentration of 700–1200 cells μl^−1^ (Single Cell 3′ library and Gel Bead Kit v.3). By leveraging microfluidic chip technology, single‐cell suspensions and beads containing cell barcodes are encapsulated in droplets to form single‐cell GEMs (Gel Bead in Emulsion). Within these droplets, mRNA from the cells undergoes reverse transcription to synthesize cDNA, which is subsequently used to construct a cDNA library. Qubit was used for library quantification before pooling. The final library pool was sequenced on the Illumina Nova6000 instrument by Shanghai Personalbio (Shanghai, China) using 150‐base‐pair paired‐end reads. Single‐cell FASTQ sequencing reads were aligned to the *D. officinale* Kimura et Migo reference genome (Niu et al., 2021). The raw data were processed using the 10x Genomics Cell Ranger software (version 3.1.0) (http://support.10xgenomics.com/single‐cell/software/overview/welcome), converting it into FASTQ files and performing counting quantification and alignment.

### Selection and clustering of dimensions in scRNA‐seq data

Before analyzing the scRNA‐seq data, we performed standard preprocessing steps for gene filtering, primarily excluding genes expressed in three or fewer cells. For cell filtering, we removed cells with a total gene count greater than 5000 or less than 400, as well as cells with a mitochondrial gene proportion exceeding 20%. We used the Harmony (1.20) algorithm for integrating multiple samples of single‐cell sequencing data, initially applying PCA for dimensionality reduction, where Harmony (1.20) iteratively removes batch effects in the PCA space. The algorithm parameters of Harmony (1.20) are set to the default settings. The raw count matrix was clustered using the Seurat package (v3.2.0) in R (v4.0.2) (Butler et al., 2018). We normalized the expression values using the LogNormalize method from the ‘Normalization’ function, followed by PCA for further dimensionality reduction. The standardized expression values were then utilized for PCA analysis. Clustering of the normalized data was performed using the Louvain algorithm, with the UMAP method employed to visualize the cell clustering results.

### Differentially expressed genes and functional enrichment analysis

For each cluster, we analyzed the DEGs within the cluster compared with other clusters using the Wilcox algorithm, selecting genes that were upregulated within the cluster. The selection criteria included: expression proportion in the cell group pct.1 or pct.2 >0.25; average log fold change avg_log2FC >0.25; and *P*‐value <0.01. We conducted pairwise differential analysis of cells from different samples within the same cluster, with the filtering criteria of |avg_log2FC| >1 and pct.1 or pct.2 >0.25. Finally, we performed GO and KEGG enrichment analyses on the selected DEGs using clusterProfiler (version: 3.18.0) (Yu et al., 2012).

### Selection and identification of marker genes

To identify the different cell types at the base of *D. officinale* stems, we annotated the cell types using known marker genes from rice. By performing a clustering analysis of the single‐copy homologous protein sequences from both *D. officinale* and rice, we determined the marker genes for each cell type. To identify the various cell types in the stem base of *D. officinale*, we annotated these cell types using known marker genes from the stems of *A. thaliana* and rice. By analyzing the single‐copy homologous protein sequences from *D. officinale*, *A. thaliana*, and rice, we determined the marker genes specific to each cell type. The protein sequences were downloaded from scPlantDB (https://biobigdata.nju.edu.cn/scplantdb/marker) relevant websites.

### Validation of marker genes by fluorescence *in situ* hybridization

To validate scRNA‐seq‐identified marker genes, FISH was performed. Gene‐specific antisense probes were labeled with digoxigenin.

*Dendrobium officinale* stems fixed in RNA‐free FAA were paraffin‐embedded and sectioned at 4 μm. After deparaffinization, rehydration, and proteinase K digestion (1 μg ml^−1^, 37°C, 20 min), sections were post‐fixed in 4% PFA and acetylated. Hybridization was performed with 500 ng ml^−1^ labeled probes (Primer 5) at 65°C for 48 h, followed by stringent washes (formamide: 4× SSC, 60°C–65°C). Signals were visualized using a Pannoramic MIDI II scanner.

### Pseudotime trajectory analysis

Monocle version 2.0 is one of the most widely used methods for single‐cell pseudotime analysis. It enables differential analysis and pseudotime analysis of cells, integrating clustering and visualization of the results. We identified key genes associated with developmental and differentiation processes and grouped genes with similar expression trends for KEGG/GO enrichment analysis.

### Bulk RNA‐seq

Healthy 1‐year‐old *D. officinale* tissue culture seedlings were selected as experimental materials. A 5 mm *S. rolfsii* plug was inoculated at the base of each seedling. Samples from the base of the seedlings were collected at 0, 12, and 24 h post‐treatment, with three independent replicates for each time point, and immediately frozen in liquid nitrogen for storage at −80°C until use. Total RNA was extracted from these samples using the EASY Spin Plant RNA Kit (Aidlab, Beijing, China). RNA sequencing was performed on a high‐throughput sequencing platform, Illumina NovaSeq, and the sequencing data were filtered using Fastp. The filtered reads were aligned to the *D. officinale* genome using HISAT2 (http://ccb.jhu.edu/software/hisat2/index.shtml). HTSeq was used to count the number of reads mapped to each gene, providing the raw expression levels, which were then normalized using FPKM. Differential expression analysis was conducted with DESeq (Love et al., 2014), applying the criteria of |log2FoldChange| >1 and a significance threshold of *P*‐value <0.05 to filter DEGs (Varet et al., 2016).

### Weighted gene co‐expression network analysis

DEGs in the CK, T_12h, and T_24h groups from the transcriptomic data were analyzed using WGCNA. It is assumed that genes with related functions may exhibit similar expression patterns (Langfelder & Horvath, 2008). The parameters were as follows: minModuleSize: 30, maxBlockSize: 2000, and deepSplit: 2.

### Identification of transcription factor protein sequences in the *D. officinale* genome

To identify candidate members of the biotic stress‐related TF gene family in the *D. officinale* genome, the Hidden Markov Model (HMMs) map of *bZIP* (PF00170, PF03131, and PF07716), *HSP90* (PF00183), *WRKY* (PF03106), *VQ* (PF05678), *BES1* (PF05687), *NBS* (PF00931, PF01582, PF00560, PF07723, PF07725, PF12799, PF13306, PF08191, PF13855, and PF05659), *MYB* (PF00249), and *bHLH* (PF00010) was downloaded from the Protein family database (PFam) (https://pfam.xfam.org/). All candidate sequences were submitted to Pfam (https://pfam.xfam.org/), SMART (http://smart.embl‐heidelberg.de/), and NCBI‐CDD3 (https://www.ncbi.nlm.nih.gov/Structure/cdd/wrpsb.cgi) to eliminate false‐positives. Using TBtools software, a heatmap was constructed based on normalized gene expression data (Chen et al., 2020).

### Transcription factor overexpression analysis

Full‐length cDNA of *HSP81*, *WRKY33*, and *MYB78* was amplified by PCR and cloned into the pCAMBIA1301‐35SN binary vector. The successfully ligated binary vector was transformed into *Agrobacterium tumefaciens* GV3101 using the electroporation method. The *Agrobacterium*‐mediated transformation vector was then introduced into *D. officinale* leaves, followed by a 12‐h dark treatment. On the third day after introduction, leaf samples were collected, and qPCR was used to measure the expression levels of the relevant genes.

### Quantitative real‐time PCR

The method for total RNA extraction was as previously described. Subsequently, cDNA synthesis was performed using HiScript® III RT SuperMix for qPCR (Vazyme, Nanjing, China). Primers were designed using Snapgene software. The quantitative reverse transcriptase‐polymerase chain reaction (qRT‐PCR) was conducted on an ABI‐7500 real‐time PCR detection system. The reaction mixture contained 1 μl template in a final volume of 20 μl, with cDNAs diluted to 200 ng. Each assay was performed in triplicate. The PCR amplification conditions were as follows: 95°C for 30 sec, followed by 40 cycles of 95°C for 10 sec and 60°C for 30 sec, with a final extension at 60°C for 15 sec. Expression data were calculated using the 2^−ΔΔCT^ method (Livak & Schmittgen, 2001). GAPDH served as the internal reference gene. Primer sequences are listed in Table S10.

### Protein–protein interaction network analysis

The protein sequence relationships of the three TFs (*HSP81*, *WRKY33*, and *MYB78*) were predicted using the online tool STRING (http://string‐db.org/), and the results were visualized with Cytoscape v3.7.2 software (Shannon et al., 2003).

### Yeast two‐hybrid assay

The recombinant plasmids pGADT7‐WRKY33 (activation domain fusion) and pGBKT7‐HSP81 (binding domain fusion) were propagated in *Escherichia coli* DH5α and purified. The resulting constructs were co‐transformed into competent yeast strain AH109 using the polyethylene glycol/lithium acetate method. Transformants were selected on synthetic defined (SD) medium lacking leucine and tryptophan (SD/–Leu/–Trp) and incubated at 30°C. To assess PPIs, positive colonies were subsequently spotted onto SD medium lacking adenine, histidine, leucine, and tryptophan (SD/–Ade/–His/–Leu/–Trp) and grown at 30°C.

### Yeast one‐hybrid

A Y1H assay was utilized to examine the binding and interaction between the TF *WRKY33* and the promoter regions of the *C4H*, *HCT*, and *4CL* genes. The protein‐coding sequence of *WRKY33* was initially cloned into the pB42AD vector using the EcoRI and XhoI restriction sites. To obtain the promoter sequences of *C4H*, *HCT*, and *4CL*, 2000 bp fragments from each gene were extracted and inserted into the pLacZi vector. The recombinant pB42AD and pLacZi plasmids were then co‐transformed into the yeast strain EJ48, and the interaction was analyzed using the X‐gal colorimetric assay. For the control group, empty vectors containing only pLacZi and pB42AD were employed to exclude non‐specific binding and background interference, thereby ensuring the reliability of the results.

### Electrophoretic mobility shift assay

The GST‐WRKY33 fusion protein was expressed in *E. coli* strain BL21 (DE3) and purified using glutathione‐Sepharose beads. EMSA was performed with a Chemiluminescent EMSA Kit (Beyotime, Shanghai, China). Biotin‐labeled probes were incubated with purified GST‐WRKY33, and protein–DNA complexes were resolved on 6% native PAGE, transferred to nylon membranes, and detected via chemiluminescence.

### Subcellular localization

Full‐length cDNA of *HSP81*, *WRKY33*, and *MYB78* was amplified by PCR, excluding the stop codons, and then individually cloned into the plant expression vector pCambia1300 to construct fusion constructs. The empty vector and fusion plasmids were subsequently expressed in tobacco leaves. Tobacco (*Nicotiana tabacum*) plants were first grown in the dark for 12 h and then infiltrated for 3 days, after which GFP fluorescence was observed using a confocal microscope.

### Phloroglucinol‐HCl staining

Cross‐sections of 1‐year‐old *D. officinale* stems, both *WRKY33* overexpressing and control group, were sliced and stained with Wiesner's reagent (paraphenylenediamine‐HCl).

### Lignin content assay

Total lignin content was measured using the acetyl bromide method. Oven‐dried (45°C, constant weight) and ground *D. officinale* stems were used. Approximately 15 mg of sample was mixed with 1 ml of 25% (v/v) acetyl bromide and 40 μl of perchloric acid, vortexed, and incubated at 80°C for 40 min (vortexing every 10 min). A blank control (without sample) was prepared in parallel. After cooling to room temperature, 400 μl of 2 m NaOH and 70 μl of 0.5 m hydroxylamine hydrochloride were added sequentially and vortexed. A 10 μl aliquot of the supernatant was mixed with 1.99 ml of glacial acetic acid, and the absorbance was measured at 280 nm using a quartz cuvette. Net absorbance (Δ*A*) was calculated as *A*_sample_ − *A*_blank_. Lignin content (mg g^−1^ dry weight) was calculated as 0.0735 × (Δ*A* − 0.0068)/(*W* × *T*), where *W* is sample mass (g) and *T* is the dilution factor.

## AUTHOR CONTRIBUTIONS

XD and LL designed the study. LL and YX performed the experiments. LL analyzed the data. XD, QX, WL, ZN, and LL collected the materials. LL wrote the manuscript. All authors approved the final version of the manuscript.

## CONFLICT OF INTEREST

The authors declare that they have no conflicts of interest.

## Supporting information


**Figure S1.** Protoplasts isolated from the stems of *D. officinale* in CK, 12 hpi, and 24 hpi samples.
**Figure S2.** The UMAP plot shows the expression of representative cluster‐specific marker genes in the stem base of *D. officinale*.
**Figure S3.** The UMAP plot shows the expression of representative differentially expressed transcription factor genes in the stem base of *D. officinale*.
**Figure S4.** WGCNA of *D. officinale* genes in *S. rolfsii* treatment.
**Table S1.** Statistical table of sequencing.
**Table S2.** Statistical table of cell counts in each subpopulation of each sample.
**Table S3.** List of cell‐type marker genes used in this study.
**Table S4.** Specific information on six different cell types.
**Table S5.** The number of differentially expressed genes in six cell types from the CK, 12 hpi, and 24 hpi samples.
**Table S6.** Statistical analysis of differentially expressed genes in six cell types between CK and 12 hpi and CK and 24 hpi.
**Table S7.** GO enrichment analysis of upregulated and downregulated differentially expressed genes in six cell types between CK and 12 hpi and CK and 24 hpi.
**Table S8.** KEGG enrichment analysis of upregulated and downregulated differentially expressed genes in six cell types between CK and 12 hpi and CK and 24 hpi.
**Table S9.** The list of WGCNA.
**Table S10.** Primers used in qRT‐PCR.
**Table S11.** The protein–protein interaction (PPI) network of three transcription factors (HSP81, WRKY33, and MYB78) in *D. officinale*.

## Data Availability

All of the raw sequence reads used in this study have been deposited in NCBI (BioProject accession numbers: PRJNA1210824 and PRJNA1212309). For more information, please visit the following websites: https://www.ncbi.nlm.nih.gov/bioproject/PRJNA1210824 and https://www.ncbi.nlm.nih.gov/bioproject/PRJNA1212309.
