# Supplementary material for: Single‐cell transcriptome atlas reveals a WRKY33 ‐ HSP81 regulatory module that orchestrates phenylpropanoid‐mediated defense against Sclerotium rolfsii  in Dendrobium officinale
Source: Plant J. 2026 Oct 29;128(3):e71190. doi: 10.1111/tpj.71190 (PMC13720222; doi:10.1111/tpj.71190)
Supplement: Supplementary file 1 [file TPJ-128-0-s001.docx]

| **Table S1 Statistical table of sequencing** |  |  |  |
| --- | --- | --- | --- |
|  | **CK** | **T_12h** | **T_24h** |
| **Estimated Number of Cells** | 9,136 | 6,355 | 8,375 |
| **Mean Reads per Cell** | 35,416 | 55,984 | 41,984 |
| **Median Genes per Cell** | 2,160 | 1,672 | 1,573 |
| **Number of Reads** | 323,564,504 | 355,777,564 | 351,618,393 |
| **Valid Barcodes** | 95.30% | 94.40% | 90.60% |
| **Valid UMIs** | 99.90% | 99.90% | 99.90% |
| **Sequencing Saturation** | 31.80% | 35.80% | 23.70% |
| **Q30 Bases in Barcode** | 96.90% | 97.20% | 97.00% |
| **Q30 Bases in RNA Read** | 95.20% | 94.90% | 94.70% |
| **Q30 Bases in UMI** | 97.00% | 97.20% | 96.90% |
| **Reads Mapped to Genome** | 88.80% | 86.30% | 85.70% |
| **Reads Mapped Confidently to Genome** | 80.80% | 68.90% | 64.40% |
| **Reads Mapped Confidently to Intergenic Regions** | 34.50% | 33.50% | 34.20% |
| **Reads Mapped Confidently to Intronic Regions** | 12.50% | 10.80% | 8.70% |
| **Reads Mapped Confidently to Exonic Regions** | 33.80% | 24.50% | 21.50% |
| **Reads Mapped Confidently to Transcriptome** | 43.70% | 33.20% | 27.90% |
| **Reads Mapped Antisense to Gene** | 2.60% | 2.20% | 2.40% |
| **Estimated Number of Cells** | 9,136 | 6,355 | 8,375 |
| **Fraction Reads in Cells** | 63.70% | 74.90% | 53.50% |
| **Mean Reads per Cell** | 35,416 | 55,984 | 41,984 |
| **Median UMI Counts per Cell** | 5,062 | 4,586 | 3,442 |
| **Median Genes per Cell** | 2,160 | 1,672 | 1,573 |
| **Total Genes Detected** | 22,761 | 23,079 | 22,487 |

| **Table S2 Statistical table of cell counts in each subpopulation of each sample** | | | | | | | | | | | | | | | | | | | | | | |
| --- | --- | --- | --- | --- | --- | --- | --- | --- | --- | --- | --- | --- | --- | --- | --- | --- | --- | --- | --- | --- | --- | --- |
| **sample** | **0** | **1** | **2** | **3** | **4** | **5** | **6** | **7** | **8** | **9** | **10** | **11** | **12** | **13** | **14** | **15** | **16** | **17** | **18** | **19** | **20** | **Total** |
| **CK** | 826 | 595 | 659 | 654 | 1055 | 268 | 546 | 343 | 412 | 581 | 313 | 442 | 574 | 329 | 264 | 411 | 216 | 218 | 126 | 150 | 123 | 9105 |
| **T_12h** | 550 | 1378 | 815 | 402 | 172 | 298 | 113 | 278 | 114 | 175 | 389 | 165 | 79 | 253 | 201 | 105 | 62 | 161 | 67 | 50 | 26 | 5853 |
| **T_24h** | 1458 | 598 | 993 | 692 | 515 | 884 | 617 | 575 | 421 | 124 | 170 | 230 | 123 | 175 | 183 | 132 | 270 | 94 | 73 | 24 | 22 | 8373 |
| **Total** | 2834 | 2571 | 2467 | 1748 | 1742 | 1450 | 1276 | 1196 | 947 | 880 | 872 | 837 | 776 | 757 | 648 | 648 | 548 | 473 | 266 | 224 | 171 |  |

| **Table S3 List of cell-type marker genes used in this study** | | | |
| --- | --- | --- | --- |
| **Cell type** | D. officinale's marker genes | O. sativa's marker genes | A. thaliana's marker genes |
| Phloem | *Dof018315* | *Os03g0743500* | *AT1G66410* |
|  |  | *Os05g0491100* | *AT1G01300* |
|  |  | *Os01g0810300* |  |
|  | *Dof023383* | *Os07g0160100* | *AT1G08465* |
| Xylem | *Dof016664* | *Os03g0726100* | *AT1G04820* |
|  |  | *Os11g0247300* | *AT1G04820* |
|  |  | *Os07g0574800* |  |
|  |  | *Os01g0282800* |  |
|  |  | *Os06g0671900* |  |
|  | *Dof019373* | *Os03g0726100* | *AT1G04820* |
|  |  | *Os11g0247300* |  |
|  |  | *Os07g0574800* |  |
|  |  | *Os01g0282800* |  |
|  |  | *Os01g0805900* |  |
| Epidermal cells | *Dof009146* | *Os01g0726700* | *AT2G42840* |
|  | *Dof001784* | *Os03g0220100* | *AT1G68530* |
|  |  | *Os05g0568000* |  |
|  |  | *Os02g0205500* |  |
|  |  | *Os03g0245700* |  |
|  |  | *Os11g0591200* |  |
|  | *Dof022073* | *Os03g0181500* | *AT2G26250* |
|  |  | *Os05g0568000* |  |
|  |  | *Os03g0245700* |  |
|  |  | *Os03g0220100* |  |
| Parenchyma cells (Vascular bundle) | *Dof012534* | *Os01g0135700* | *AT1G09340* |
|  |  | *Os03g0310800* |  |
|  | *Dof011034* | *Os04g0482300* |  |
|  |  | *Os01g0908200* |  |
| Proliferating cells | *Dof015620* | *Os07g0549900* | *AT5G59970* |
|  | *Dof003854* | *Os09g0433600* |  |
| Parenchyma cells (Ground tissue) | *Dof016958* | *Os02g0473200* |  |
|  |  | *Os02g0314600* |  |
|  | *Dof003464* | *Os03g0266900* | *AT5G59720* |
|  |  | *Os03g0266300* |  |
|  |  | *Os03g0267000* |  |

| **Table S4 Specific information on six different cell types** | | | |
| --- | --- | --- | --- |
| **cluster** | **ABV** | **cell type** | **marker genes** |
| 17 | Phloem | Phloem | *Dof018315 Dof023383* |
| 19 | Xylem | Xylem | *Dof016664 Dof019373* |
| 13 | EC | Epidermal cells | *Dof009146 Dof001784 Dof022073* |
| 1,2,5,9,14 | PV | Parenchyma cells (Vascular bundle) | *Dof012534 Dof011034* |
| 15 | PC | Proliferating cells | *Dof015620 Dof003854* |
| 0,3,4,6,7,8,10,11,12,16,18,20 | PG | Parenchyma cells (Ground tissue) | *Dof016958 Dof003464* |

| **Table S5: The number of differentially expressed genes in six cell types from the CK, 12hpi, and 24hpi samples.** | | | | | | | |
| --- | --- | --- | --- | --- | --- | --- | --- |
|  | **Phloem** | **Xylem** | **EC** | **PV** | **PC** | **PG** | **total** |
| **CK** | 218(2.39%) | 150(1.65%) | 329(3.61%) | 2367(26.01%) | 411(4.51%) | 5630(61.83%) | 9105(100%) |
| **T_12h** | 161(2.75%) | 50(0.85%) | 253(4.32%) | 2867(48.99%) | 105(1.79%) | 2417(41.30%) | 5853(100%) |
| **T_24h** | 94(1.12%) | 24(0.29%) | 175(2.08%) | 2782(33.23%) | 132(1.58%) | 5166(61.70%) | 8373(100%) |

| **Table S6: Statistical analysis of differentially expressed genes in six cell types between CK&12hpi and CK&24hpi.** | | | | | | |
| --- | --- | --- | --- | --- | --- | --- |
|  | **Phloem** | **Xylem** | **EC** | **PV** | **PC** | **PG** |
| **CK&T_12h** | Dof012506 | Dof001708 | Dof012504 | Dof000351 | Dof005749 | Dof000380 |
|  | Dof012507 | Dof013391 | Dof012507 | Dof000496 | Dof001708 | Dof000496 |
|  | Dof024912 | Dof005749 | Dof012506 | Dof000497 | Dof012504 | Dof000785 |
|  | Dof001894 | Dof019832 | Dof002316 | Dof001825 | Dof013847 | Dof001825 |
|  | Dof004391 | Dof021077 | Dof002923 | Dof002316 | Dof012506 | Dof002290 |
|  | Dof022876 | Dof008472 | Dof017284 | Dof002704 | Dof012507 | Dof002316 |
|  | Dof014544 | Dof024575 | Dof019832 | Dof003271 | Dof021077 | Dof002704 |
|  | Dof001708 | Dof006415 | Dof014007 | Dof003326 | Dof016826 | Dof003974 |
|  | Dof003974 | Dof002112 | Dof015826 | Dof003819 | Dof023703 | Dof003990 |
|  | Dof002923 | Dof013847 | Dof024575 | Dof003990 | Dof014544 | Dof003999 |
|  | Dof015798 | Dof018248 | Dof012263 | Dof003999 | Dof014294 | Dof004018 |
|  | Dof003361 | Dof017285 | Dof001894 | Dof004018 | Dof018248 | Dof004216 |
|  | Dof000767 | Dof011195 | Dof016826 | Dof004175 | Dof024575 | Dof004416 |
|  | Dof012210 | Dof021078 | Dof014922 | Dof004207 | Dof013391 | Dof004610 |
|  | Dof008403 | Dof009338 | Dof006539 | Dof004416 | Dof022876 | Dof004829 |
|  | Dof001521 | Dof004334 | Dof007001 | Dof004829 | Dof012677 | Dof005148 |
|  | Dof017284 | Dof001894 | Dof022876 | Dof005148 | Dof009721 | Dof005700 |
|  | Dof013874 | Dof003974 | Dof014177 | Dof005700 | Dof013874 | Dof005749 |
|  | Dof020861 | Dof016900 | Dof024361 | Dof005870 | Dof014645 | Dof005870 |
|  | Dof014007 | Dof018067 | Dof024912 | Dof006253 | Dof002316 | Dof006047 |
|  | Dof016826 | Dof023703 | Dof009820 | Dof006415 | Dof021278 | Dof006217 |
|  | Dof013349 | Dof006598 | Dof022873 | Dof006582 | Dof017423 | Dof006253 |
|  | Dof009322 | Dof018844 | Dof013652 | Dof007001 | Dof023143 | Dof006361 |
|  | Dof012504 | Dof009227 | Dof015798 | Dof007002 | Dof000695 | Dof006415 |
|  | Dof007734 | Dof007001 | Dof024119 | Dof007590 | Dof019290 | Dof007001 |
|  | Dof005749 | Dof014544 | Dof021077 | Dof007952 | Dof006415 | Dof007002 |
|  | Dof021077 | Dof014788 | Dof011043 | Dof008010 | Dof017150 | Dof007323 |
|  | Dof013652 | Dof013666 | Dof022092 | Dof008070 | Dof020523 | Dof007590 |
|  | Dof022255 | Dof013874 | Dof006415 | Dof009492 | Dof001693 | Dof008010 |
|  | Dof015826 | Dof000920 | Dof007002 | Dof009814 | Dof013666 | Dof008472 |
|  | Dof000522 | Dof017564 | Dof005749 | Dof010192 | Dof004901 | Dof009152 |
|  | Dof013847 | Dof024522 | Dof004138 | Dof010802 | Dof019832 | Dof009814 |
|  | Dof010115 | Dof012677 | Dof016820 | Dof011163 | Dof003974 | Dof010802 |
|  | Dof018067 | Dof007002 | Dof003254 | Dof011195 | Dof020859 | Dof011027 |
|  | Dof009820 | Dof025361 | Dof013847 | Dof011204 | Dof004391 | Dof011163 |
|  | Dof002316 | Dof004397 | Dof012502 | Dof011223 | Dof008472 | Dof011195 |
|  | Dof011043 | Dof013695 | Dof014182 | Dof011289 | Dof019447 | Dof011223 |
|  | Dof003954 | Dof004401 | Dof003974 | Dof011509 | Dof015826 | Dof011289 |
|  | Dof000497 | Dof017805 | Dof000767 | Dof011987 | Dof009472 | Dof011509 |
|  | Dof024810 | Dof024663 | Dof004391 | Dof012263 | Dof000380 | Dof011987 |
|  | Dof014645 | Dof004901 | Dof014544 | Dof013145 | Dof012263 | Dof012263 |
|  | Dof025255 | Dof012263 | Dof001521 | Dof013170 | Dof019408 | Dof012504 |
|  | Dof014643 | Dof020861 | Dof014524 | Dof013349 | Dof024522 | Dof012507 |
|  | Dof014570 | Dof024361 | Dof019290 | Dof013847 | Dof002554 | Dof012612 |
|  | Dof017636 | Dof014294 | Dof022377 | Dof014001 | Dof003184 | Dof012677 |
|  | Dof004901 | Dof020454 | Dof017312 | Dof014524 | Dof017805 | Dof013145 |
|  | Dof017285 | Dof014645 | Dof022904 | Dof014542 | Dof006598 | Dof013146 |
|  | Dof006539 | Dof022198 | Dof011996 | Dof014543 | Dof018577 | Dof013847 |

|  | **Phloem** | **Xylem** | **EC** | **PV** | **PC** | **PG** |
| --- | --- | --- | --- | --- | --- | --- |
| **CK&T_24h** | Dof024300 | Dof024300 | Dof024575 | Dof000384 | Dof024300 | Dof000132 |
|  | Dof014546 | Dof004610 | Dof017968 | Dof000767 | Dof004610 | Dof000232 |
|  | Dof021077 | Dof007323 | Dof024300 | Dof001729 | Dof014546 | Dof000286 |
|  | Dof013874 | Dof020105 | Dof014546 | Dof001960 | Dof021077 | Dof000351 |
|  | Dof016529 | Dof024575 | Dof021077 | Dof002316 | Dof013874 | Dof000384 |
|  | Dof017512 | Dof016351 | Dof024912 | Dof003326 | Dof012505 | Dof000391 |
|  | Dof003999 | Dof021077 | Dof007002 | Dof003999 | Dof024912 | Dof000496 |
|  | Dof024912 | Dof015957 | Dof024119 | Dof004018 | Dof018963 | Dof000518 |
|  | Dof017968 | Dof016529 | Dof012263 | Dof004122 | Dof016529 | Dof000533 |
|  | Dof007002 | Dof003999 | Dof004610 | Dof004175 | Dof024575 | Dof000879 |
|  | Dof013349 | Dof001729 | Dof016529 | Dof004238 | Dof012263 | Dof001150 |
|  | Dof008076 | Dof024912 | Dof012612 | Dof004610 | Dof007002 | Dof001214 |
|  | Dof024575 | Dof015082 | Dof008076 | Dof005320 | Dof024119 | Dof001402 |
|  | Dof019740 | Dof024646 | Dof007001 | Dof005700 | Dof007590 | Dof001553 |
|  | Dof017285 | Dof017285 | Dof015082 | Dof005715 | Dof017285 | Dof001676 |
|  | Dof022972 | Dof005749 | Dof016632 | Dof006415 | Dof017968 | Dof001729 |
|  | Dof011043 | Dof012505 | Dof006415 | Dof007002 | Dof007323 | Dof001745 |
|  | Dof003084 | Dof008472 | Dof022972 | Dof007590 | Dof024360 | Dof001924 |
|  | Dof001156 | Dof011195 | Dof010278 | Dof008070 | Dof023258 | Dof001965 |
|  | Dof010278 | Dof024360 | Dof014642 | Dof008076 | Dof008076 | Dof002290 |
|  | Dof007590 | Dof014546 | Dof017285 | Dof009322 | Dof016351 | Dof002316 |
|  | Dof002923 | Dof007326 | Dof015798 | Dof009378 | Dof015082 | Dof002461 |
|  | Dof024109 | Dof019929 | Dof017564 | Dof009459 | Dof000384 | Dof002577 |
|  | Dof000384 | Dof017564 | Dof021174 | Dof010192 | Dof019931 | Dof002588 |
|  | Dof013042 | Dof013229 | Dof011195 | Dof010278 | Dof010278 | Dof002598 |
|  | Dof015308 | Dof017126 | Dof007326 | Dof011289 | Dof025256 | Dof002704 |
|  | Dof024119 | Dof021078 | Dof020923 | Dof011509 | Dof008472 | Dof002907 |
|  | Dof025255 | Dof018963 | Dof017239 | Dof012263 | Dof002577 | Dof002923 |
|  | Dof014823 | Dof013874 | Dof025361 | Dof012505 | Dof011195 | Dof003018 |
|  | Dof012263 | Dof002290 | Dof022405 | Dof012612 | Dof004238 | Dof003084 |
|  | Dof014552 | Dof021891 | Dof002923 | Dof013042 | Dof014524 | Dof003251 |
|  | Dof015798 | Dof007002 | Dof004400 | Dof013125 | Dof007326 | Dof003276 |
|  | Dof000232 | Dof004292 | Dof014073 | Dof013349 | Dof025255 | Dof003326 |
|  | Dof009322 | Dof017968 | Dof019931 | Dof014001 | Dof025361 | Dof003791 |
|  | Dof013125 | Dof024437 | Dof007590 | Dof014007 | Dof014544 | Dof003854 |
|  | Dof019931 | Dof012263 | Dof017788 | Dof014544 | Dof006717 | Dof003951 |
|  | Dof014544 | Dof018067 | Dof014177 | Dof014546 | Dof001729 | Dof003974 |
|  | Dof014193 | Dof014683 | Dof006539 | Dof014678 | Dof012612 | Dof003990 |
|  | Dof010744 | Dof013125 | Dof008472 | Dof014759 | Dof006415 | Dof003999 |
|  | Dof018067 | Dof025361 | Dof014524 | Dof014786 | Dof022405 | Dof004018 |
|  | Dof004610 | Dof014330 | Dof006047 | Dof015366 | Dof015308 | Dof004106 |
|  | Dof000767 | Dof002923 | Dof019832 | Dof015599 | Dof015798 | Dof004137 |
|  | Dof020923 | Dof016246 | Dof013349 | Dof015798 | Dof017564 | Dof004173 |
|  | Dof024810 | Dof000380 | Dof013264 | Dof016529 | Dof013125 | Dof004175 |
|  | Dof017788 | Dof004238 | Dof000384 | Dof016724 | Dof001960 | Dof004207 |
|  | Dof005320 | Dof008076 | Dof024360 | Dof017285 | Dof004746 | Dof004238 |
|  | Dof024360 | Dof006496 | Dof005387 | Dof017304 | Dof014922 | Dof004292 |
|  | Dof000763 | Dof001368 | Dof006892 | Dof017636 | Dof020923 | Dof004397 |
|  | Dof015082 | Dof001402 | Dof014544 | Dof017788 | Dof003999 | Dof004400 |

| **Table S7: GO enrichment analysis of upregulated and downregulated differentially expressed genes in six cell types between CK&12hpi and CK&24hpi.** | | | | | | | |
| --- | --- | --- | --- | --- | --- | --- | --- |
| ck&12h up |  | **PG** | **PC** | **Xylem** | **PV** | **Phloem** | **EC** |
| GO:0009607 | response to biotic stimulus | 0.572216838 | 1.170924493 | 1.514197761 | 1.594708282 | 0.881708247 | 1.277220523 |
| GO:0006952 | defense response | 0.515918671 | 0.956060518 | 0.93984716 | 0.411665345 | 1.720924147 | 1.932025184 |
| GO:0008299 | isoprenoid biosynthetic process | 0.281723343 | 0.488257289 | 0.640679146 | 0 | 0.82840809 | 0.535128877 |
| GO:0006032 | chitin catabolic process | 0.112851832 | 0.028718127 | 0.052016712 | 0.572864325 | 0.340128804 | 0.36549355 |
| GO:0016998 | cell wall macromolecule catabolic process | 0.203122677 | 0.087818897 | 0.125341291 | 0.380657536 | 0.521733485 | 0.546794173 |
| GO:0005975 | carbohydrate metabolic process | 0.104224824 | 0.092119589 | 0.009050655 | 0.678775799 | 0.040366978 | 0.234401395 |
| GO:0006457 | protein folding | 0.626058596 | 0.444892964 | 0.402287958 | 1.797703699 | 0.48598502 | 0.050857318 |
| GO:0050832 | defense response to fungus | 3.429443197 | 0.653238858 | 1.024359481 | 1.575033557 | 2.547763027 | 2.03625667 |
| GO:0006096 | glycolytic process | 3.848264582 | 2.45626477 | 3.292837757 | 0.848181009 | 3.328473345 | 2.356182789 |
| GO:0042545 | cell wall modification | 0.353258567 | 0.140749107 | 0.254214353 | 0.535255799 | 0.153152725 | 0.0992826 |
| GO:0006979 | response to oxidative stress | 0.260662622 | 0.007898429 | 0.002946549 | 0.346254731 | 0.22004533 | 0.169284373 |
| GO:0034599 | cellular response to oxidative stress | 1.011214733 | 0.918738759 | 0.005551219 | 0.927106331 | 0.965008843 | 0.172673254 |
| GO:0042542 | response to hydrogen peroxide | 1.504020015 | 0.792988737 | 0.47911999 | 1.879166889 | 0.013932961 | 0.344580921 |
| GO:0050896 | response to stimulus | 0 | 0.137690861 | 0.06941159 | 0 | 0.047229409 | 0.051636019 |
| GO:1900150 | regulation of defense response to fungus | 2.709427702 | 0.792988737 | 1.450977364 | 0 | 1.080549471 | 1.778790493 |

| ck&12h down |  | **PG** | **PC** | **Xylem** | **PV** | **Phloem** | **EC** |
| --- | --- | --- | --- | --- | --- | --- | --- |
| GO:0009607 | response to biotic stimulus | 0.189535881 | 0 | 0 | 0.056265958 | 0 | 0 |
| GO:0006952 | defense response | 0.237501988 | 0.408888524 | 0.905207876 | 0.834087936 | 0.350615631 | 0.34636022 |
| GO:0008299 | isoprenoid biosynthetic process | 0.213931926 | 0 | 0.203876835 | 0.811797567 | 0.183600197 | 0.146577128 |
| GO:0006032 | chitin catabolic process | 0.251342759 | 0.140351564 | 0 | 0.216583027 | 0.105844838 | 0 |
| GO:0016998 | cell wall macromolecule catabolic process | 0.741908611 | 0.310797835 | 0 | 0.682658404 | 0.259612086 | 0 |
| GO:0005975 | carbohydrate metabolic process | 0.04355135 | 0.00674721 | 0.007108442 | 0.012747201 | 0.051939525 | 0.043956589 |
| GO:0006457 | protein folding | 2.950499694 | 0.956113759 | 1.085760695 | 2.585812179 | 0.670848937 | 0.885446771 |
| GO:0050832 | defense response to fungus | 0.372702207 | 0.41906091 | 0.533394049 | 0.244159104 | 0.393098606 | 0.262999228 |
| GO:0006096 | glycolytic process | 0.807026936 | 0.171789849 | 0.767677366 | 0.486777393 | 0.41777512 | 1.736422744 |
| GO:0042545 | cell wall modification | 0.010120712 | 0 | 0.032129786 | 0.007114956 | 0 | 0.015311288 |
| GO:0006979 | response to oxidative stress | 0.154005555 | 0.373127455 | 0.709398298 | 0.190311978 | 0.554674918 | 0.217045999 |
| GO:0034599 | cellular response to oxidative stress | 1.096016721 | 1.556229556 | 1.39203489 | 0.285819308 | 1.72363649 | 2.391375679 |
| GO:0042542 | response to hydrogen peroxide | 0.716138851 | 1.990871993 | 1.791644082 | 0.407071049 | 0.792476652 | 0.355814267 |
| GO:0050896 | response to stimulus | 0.970275303 | 0.245755236 | 0.220670446 | 0.891050586 | 0.199626141 | 0.505146697 |
| GO:1900150 | regulation of defense response to fungus | 0.484989426 | 0 | 0.120931805 | 0.865750434 | 0.099523036 | 0.175631288 |

| ck&24h up |  | **PG** | **PC** | **Xylem** | **PV** | **Phloem** | **EC** |
| --- | --- | --- | --- | --- | --- | --- | --- |
| GO:0009607 | response to biotic stimulus | 2.474033804 | 1.310980935 | 2.807600024 | 2.357169815 | 2.977432097 | 0.665414918 |
| GO:0006952 | defense response | 4.435183652 | 4.912596603 | 3.269787216 | 8.466495931 | 5.505495416 | 4.363511218 |
| GO:0006032 | chitin catabolic process | 0.561922578 | 1.483532687 | 2.169124931 | 1.296087932 | 1.685540265 | 0.746761018 |
| GO:0016998 | cell wall macromolecule catabolic process | 0.609230075 | 0.872188962 | 2.176356026 | 1.465507328 | 1.78778066 | 0.474005091 |
| GO:0005975 | carbohydrate metabolic process | 0.153464882 | 0.225470843 | 0.129697773 | 0.067660519 | 0.55090655 | 0.132642967 |
| GO:0006457 | protein folding | 1.027617598 | 3.027525522 | 2.517134202 | 2.959536449 | 0.912244585 | 0.748894707 |
| GO:0050832 | defense response to fungus | 7.461866647 | 6.307466749 | 1.52939912 | 3.110108658 | 4.182492173 | 4.44654305 |
| GO:0006096 | glycolytic process | 2.936122784 | 1.710774708 | 0.447424239 | 2.811813991 | 0.468612544 | 0.451247365 |
| GO:0042545 | cell wall modification | 0.912006805 | 1.095417691 | 0.112785682 | 1.319947622 | 0.486857793 | 1.259384351 |
| GO:0006979 | response to oxidative stress | 0.943017168 | 1.731074604 | 0.30561657 | 1.023498106 | 1.931102467 | 1.973662131 |
| GO:0034599 | cellular response to oxidative stress | 0.06244663 | 0.165925977 | 0 | 0.560030694 | 0.219350442 | 0.113826489 |
| GO:0042542 | response to hydrogen peroxide | 2.628808022 | 8.193966324 | 3.946662968 | 4.151146442 | 4.471529579 | 2.432533786 |
| GO:1900150 | regulation of defense response to fungus | 2.628808022 | 1.029203294 | 0.714881617 | 0.272497412 | 0.453620571 | 0.719560679 |

| ck&24h down |  | **PG** | **PC** | **Xylem** | **PV** | **Phloem** | **EC** |
| --- | --- | --- | --- | --- | --- | --- | --- |
| GO:0009607 | response to biotic stimulus | 0.126416352 | 0.007040404 | 0 | 0.082199555 | 0 | 0 |
| GO:0006952 | defense response | 0.055147602 | 0.013687488 | 0.004351243 | 0.05278534 | 0.013276624 | 0.009250313 |
| GO:0008299 | isoprenoid biosynthetic process | 0.382861927 | 0.37280987 | 0.670827705 | 0.959883166 | 0.617416352 | 0.835492694 |
| GO:0006032 | chitin catabolic process | 0.062604144 | 0.059763688 | 0 | 0.112714256 | 0.045787353 | 0.028107217 |
| GO:0016998 | cell wall macromolecule catabolic process | 0.271222416 | 0.069953106 | 0 | 0.375924601 | 0.229214339 | 0.177361781 |
| GO:0005975 | carbohydrate metabolic process | 0.010003677 | 0.004804711 | 0.006829388 | 0.002755804 | 0.296612745 | 0.047532092 |
| GO:0006457 | protein folding | 3.202021639 | 2.740348351 | 1.814172065 | 2.164094672 | 2.61807025 | 4.36877212 |
| GO:0050832 | defense response to fungus | 0.218874706 | 0.149467892 | 0.035661245 | 0.107839532 | 0.022876714 | 0.001815884 |
| GO:0006096 | glycolytic process | 0.149550383 | 0.229567489 | 0.52515839 | 0.331901151 | 1.775849415 | 0.629681428 |
| GO:0042545 | cell wall modification | 0.000519971 | 0.000467588 | 0.00453654 | 0 | 0.000254403 | 0.004576036 |
| GO:0006979 | response to oxidative stress | 0.145173996 | 0.091791722 | 0.019761643 | 0.047543939 | 0.066612558 | 0.040103224 |
| GO:0034599 | cellular response to oxidative stress | 0.556902271 | 1.047814517 | 1.205692043 | 0.625764688 | 2.292138815 | 0.626666696 |
| GO:0042542 | response to hydrogen peroxide | 0.491620473 | 0.177520188 | 0.073392484 | 0.358336305 | 0.372467528 | 0.546115651 |
| GO:0050896 | response to stimulus | 1.267999856 | 0.418343098 | 0.098863545 | 1.612501029 | 0.688232159 | 0.280572792 |
| GO:1900150 | regulation of defense response to fungus | 0.31757712 | 0.039478 | 0.073392484 | 0.205667165 | 0.026138685 | 0.073053575 |

| **Table S8: KEGG enrichment analysis of upregulated and downregulated differentially expressed genes in six cell types between CK&12hpi and CK&24hpi.** | | | | | | | |
| --- | --- | --- | --- | --- | --- | --- | --- |
| **ABV** |  | **PathwayID** | **Pathway** | **List** | **Total** | **Pvalue** | **adjustPvalue** |
| EC | up | dct03015 | mRNA surveillance pathway | 52 | 137 | 0.000102165 | 0.006660811 |
|  |  | dct00592 | alpha-Linolenic acid metabolism | 32 | 73 | 0.000102474 | 0.006660811 |
|  | down | dct03010 | Ribosome | 112 | 304 | 1.14E-42 | 1.21E-40 |
| PG | up | dct03015 | mRNA surveillance pathway | 42 | 137 | 0.000182349 | 0.014016157 |
|  |  | dct00230 | Purine metabolism | 33 | 102 | 0.0002963 | 0.014016157 |
|  | down | dct03010 | Ribosome | 131 | 304 | 6.18E-35 | 7.29E-33 |
| PC | up | dct03015 | mRNA surveillance pathway | 66 | 137 | 1.88E-09 | 2.44E-07 |
|  | down | dct03010 | Ribosome | 86 | 304 | 5.11E-32 | 4.80E-30 |
| Phloem | up | dct00620 | Pyruvate metabolism | 40 | 96 | 0.00015825 | 0.010885182 |
|  |  | dct00592 | alpha-Linolenic acid metabolism | 32 | 73 | 0.000234217 | 0.010885182 |
|  |  | dct01230 | Biosynthesis of amino acids | 83 | 240 | 0.00025713 | 0.010885182 |
|  | down | dct03010 | Ribosome | 97 | 304 | 1.23E-37 | 1.18E-35 |
| PV | up | dct01200 | Carbon metabolism | 32 | 277 | 0.000359232 | 0.036641703 |
|  |  | dct00480 | Glutathione metabolism | 13 | 80 | 0.001079687 | 0.053401199 |
|  |  | dct01110 | Biosynthesis of secondary metabolites | 90 | 1101 | 0.001570623 | 0.053401199 |
|  | down | dct03010 | Ribosome | 106 | 304 | 2.63E-20 | 2.97E-18 |
| Xylem | up | dct00970 | Aminoacyl-tRNA biosynthesis | 26 | 70 | 0.001589408 | 0.104731521 |
|  |  | dct03015 | mRNA surveillance pathway | 44 | 137 | 0.001754229 | 0.104731521 |
|  |  | dct03040 | Spliceosome | 63 | 214 | 0.002454645 | 0.104731521 |
|  | down | dct03010 | Ribosome | 101 | 304 | 1.65E-41 | 1.69E-39 |

| **ABV** |  | **PathwayID** | **Pathway** | **List** | **Total** | **Pvalue** | **adjustPvalue** |
| --- | --- | --- | --- | --- | --- | --- | --- |
| EC | up | dct04626 | Plant-pathogen interaction | 21 | 203 | 4.38E-06 | 0.000332944 |
|  | down | dct03010 | Ribosome | 173 | 304 | 2.97E-44 | 3.80E-42 |
| PG | up | dct04626 | Plant-pathogen interaction | 34 | 203 | 7.08E-05 | 0.004088495 |
|  |  | dct00600 | Sphingolipid metabolism | 12 | 40 | 7.24E-05 | 0.004088495 |
|  | down | dct03010 | Ribosome | 149 | 304 | 1.22E-40 | 1.52E-38 |
| PC | up | dct04626 | Plant-pathogen interaction | 31 | 203 | 3.44E-07 | 2.39E-05 |
|  |  | dct04141 | Protein processing in endoplasmic reticulum | 35 | 249 | 4.59E-07 | 2.39E-05 |
|  | down | dct03010 | Ribosome | 164 | 304 | 4.50E-50 | 5.49E-48 |
| Phloem | up | dct04626 | Plant-pathogen interaction | 26 | 203 | 4.04E-06 | 0.000354999 |
|  |  | dct01100 | Metabolic pathways | 144 | 2265 | 7.24E-06 | 0.000354999 |
|  | down | dct03010 | Ribosome | 165 | 304 | 7.31E-45 | 9.14E-43 |
| PV | up | dct04626 | Plant-pathogen interaction | 35 | 203 | 8.59E-07 | 9.36E-05 |
|  |  | dct04141 | Protein processing in endoplasmic reticulum | 37 | 249 | 1.58E-05 | 0.000858808 |
|  | down | dct03010 | Ribosome | 106 | 304 | 9.81E-23 | 1.09E-20 |
| Xylem | up | dct04141 | Protein processing in endoplasmic reticulum | 21 | 249 | 8.37E-05 | 0.007115938 |
|  | down | dct03010 | Ribosome | 150 | 304 | 1.24E-59 | 1.41E-57 |

| Table S9 The list of WGCNA | | | | | | | |
| --- | --- | --- | --- | --- | --- | --- | --- |
| **ID** | **module** | **GS-CK** | **p.GS-CK** | **GS-T_12h** | **p.GS-T_12h** | **GS-T_24h** | **p.GS-T_24h** |
| Dof007002 | blue | 0.87837336801216 | 0.00183076729278064 | -0.851601346 | 0.0035740587454688 | -0.026772022 | 0.945493049689032 |
| Dof007001 | blue | 0.721462978019404 | 0.0282342626654115 | -0.957494154 | 5.00059930832603e-05 | 0.236031176198448 | 0.540928555556841 |
| Dof019019 | blue | -0.871242108 | 0.00221865326770408 | 0.859736638075933 | 0.00295857780772442 | 0.0115054694803014 | 0.976563835492356 |
| Dof005974 | turquoise | -0.926948552 | 0.000322836759968052 | 0.169958770104672 | 0.661989373509238 | 0.756989781569996 | 0.0181928843144549 |
| Dof013490 | brown | 0.29040313027815 | 0.448410677590817 | -0.973606027 | 9.5853139608321e-06 | 0.683202896850759 | 0.0424974216265958 |
| Dof014159 | turquoise | 0.0813207468728858 | 0.835244982596339 | 0.821483860955635 | 0.00661492765187382 | -0.902804608 | 0.000856106281036159 |
| Dof022385 | turquoise | -0.84867058 | 0.00381576229048058 | -0.03129801 | 0.936292242528834 | 0.879968589901449 | 0.00175093025272509 |
| Dof020958 | turquoise | -0.701162268 | 0.035331520373222 | -0.263929257 | 0.492568811723709 | 0.965091525789006 | 2.52908078984835e-05 |
| Dof013103 | brown | -0.611434356 | 0.0801935294084416 | 0.990494309454131 | 2.73186634920887e-07 | -0.379059954 | 0.314374510699347 |
| Dof005715 | brown | -0.178666182 | 0.645567932039362 | 0.938802078888581 | 0.000175787690481447 | -0.760135897 | 0.0174400208625688 |
| Dof001720 | blue | -0.872031965 | 0.00217312989669288 | 0.859802558208901 | 0.00295391334642926 | 0.0122294071349217 | 0.975089561179221 |
| Dof011034 | blue | -0.770077088 | 0.0151979821619836 | 0.93368842695442 | 0.000231613165383639 | -0.163611339 | 0.67403957828674 |
| Dof002548 | blue | 0.924404621735819 | 0.000363004474576515 | -0.79122262 | 0.011087233683712 | -0.133182002 | 0.732662291322103 |
| Dof022972 | blue | 0.991887739647035 | 1.57073669978417e-07 | -0.599912473 | 0.0876812690751523 | -0.391975267 | 0.296777847780614 |
| Dof014367 | turquoise | -0.693741269 | 0.0381890486707575 | -0.276767966 | 0.470934903603184 | 0.97050923449752 | 1.4090293543594e-05 |
| Dof022065 | turquoise | -0.931246575 | 0.000262225082541485 | 0.150497888601315 | 0.699136218517206 | 0.780748686057956 | 0.013014945041077 |
| Dof017788 | turquoise | 0.80537274370036 | 0.00880197820615831 | 0.109369595314476 | 0.779403101626524 | -0.914742339 | 0.00054770296093632 |
| Dof017384 | blue | -0.950682841 | 8.35643229631213e-05 | 0.73186429774517 | 0.0249913869759688 | 0.218818543730678 | 0.571632407646304 |
| Dof011099 | brown | -0.091808847 | 0.814277294113347 | 0.907791889345487 | 0.000715548826465551 | -0.815983043 | 0.00731431304522697 |
| Dof017845 | turquoise | 0.3195735495621 | 0.40186162857366 | 0.659607040073049 | 0.053246139261689 | -0.97918059 | 4.20100288667578e-06 |
| Dof008334 | turquoise | -0.848307275 | 0.00384648464770111 | -0.032407683 | 0.934037365615871 | 0.880714958099076 | 0.00171441841402296 |
| Dof019680 | turquoise | -0.289595581 | 0.449731424039603 | -0.680573514 | 0.0436189958900588 | 0.970169095499601 | 1.46624382902135e-05 |
| Dof021564 | turquoise | -0.860037684 | 0.0029373172635539 | -0.008831012 | 0.982010779202807 | 0.868868695982554 | 0.00235940456928384 |
| Dof006452 | turquoise | 0.264133069645335 | 0.492222231965036 | 0.702270091384518 | 0.0349172413038907 | -0.966403161 | 2.21467407194311e-05 |
| Dof017329 | brown | -0.39175914 | 0.29706806889017 | 0.992451255794119 | 1.22153775849506e-07 | -0.600692116 | 0.0871615603097767 |
| Dof006286 | blue | -0.768412081 | 0.0155592236585165 | 0.937916397601798 | 0.000184691498192685 | -0.169504317 | 0.662849933729859 |
| Dof019227 | brown | 0.355423863643029 | 0.347892835928459 | -0.986059282 | 1.03904430755257e-06 | 0.630635418541343 | 0.0686222249486303 |
| Dof022063 | blue | -0.8136917 | 0.00761987884784749 | 0.909670165215245 | 0.000667077663156837 | -0.095978465 | 0.805969024643916 |
| Dof003999 | turquoise | -0.541983889 | 0.131716988265563 | -0.456789391 | 0.216435683782906 | 0.998773279731034 | 2.12631874150528e-10 |
| Dof023683 | blue | 0.94670972856842 | 0.000109165787469565 | -0.748993445 | 0.0202028522585299 | -0.197716284 | 0.61010723193785 |
| Dof007796 | blue | -0.893180418 | 0.00117985488111488 | 0.835099567422784 | 0.00508222153890824 | 0.0580808506089072 | 0.882010777559926 |
| Dof020505 | blue | 0.80450123650586 | 0.00893256353198354 | -0.914332482 | 0.000556744948905082 | 0.109831245158468 | 0.778490625159188 |
| Dof001391 | blue | 0.840274298536545 | 0.00456991533119777 | -0.887443175 | 0.00140873975784655 | 0.0471688765431556 | 0.904086337590545 |
| Dof005891 | turquoise | -0.926536748 | 0.000329116040711967 | 0.138051205909701 | 0.72319269607466 | 0.788485541699361 | 0.0115710393907447 |
| Dof015260 | turquoise | 0.0225346986245674 | 0.954112111420367 | 0.854153449238259 | 0.00337233422627195 | -0.876688148 | 0.00191780993791183 |
| Dof022365 | blue | 0.637440144923449 | 0.0647887400676206 | -0.984643059 | 1.4557992678943e-06 | 0.347202913767586 | 0.359941131339571 |
| Dof024439 | blue | 0.677197855980589 | 0.0450865479885865 | -0.975304173 | 7.60756764358845e-06 | 0.298106316692487 | 0.43589755642943 |
| Dof009820 | blue | 0.851230185546157 | 0.00360406997748109 | -0.877694386 | 0.00186550066468882 | 0.0264641999607892 | 0.946119029442235 |
| Dof022092 | turquoise | -0.76572354 | 0.0161545549359154 | -0.170066196 | 0.661785999290894 | 0.935789736911717 | 0.00020735981751968 |
| Dof025052 | blue | 0.960119002639563 | 4.01104614592306e-05 | -0.718153906 | 0.0293208853373646 | -0.241965097 | 0.530493133422046 |
| Dof016025 | blue | 0.815519675416283 | 0.00737542234262907 | -0.904774657 | 0.000798478359926301 | 0.0892549814721416 | 0.819374029913072 |
| Dof003385 | turquoise | -0.004341662 | 0.991155376913996 | -0.845915792 | 0.00405296519357411 | 0.850257454808955 | 0.00368354331300695 |
| Dof021627 | blue | -0.713526889 | 0.0308856095327853 | 0.963155678335575 | 3.04911380704056e-05 | -0.24962879 | 0.517133255740542 |
| Dof013198 | turquoise | -0.818349119 | 0.00700762629388916 | -0.087700205 | 0.822479769045183 | 0.906049323895595 | 0.000762668748199555 |
| Dof010532 | blue | -0.772528991 | 0.0146762817294948 | 0.9356627199028 | 0.00020877263649137 | -0.163133729 | 0.6749489287517 |
| Dof000132 | turquoise | 0.967862395266162 | 1.89861146424826e-05 | -0.267837554 | 0.485940341769404 | -0.700024842 | 0.0357601756174303 |
| Dof008581 | turquoise | 0.854172068271003 | 0.00337089201809547 | 0.0214825665302881 | 0.956252902239265 | -0.875654635 | 0.00197258508281417 |
| Dof019908 | blue | -0.872798383 | 0.00212957865876553 | 0.858494289610151 | 0.00304743687038982 | 0.0143040934877399 | 0.97086490812666 |

| **Table S10 Primers used in qRT-PCR.** | | |
| --- | --- | --- |
| Gene name | F primer sequences (5'-3') | R primer sequences (5'-3') |
| *Dof014188* | CGCAGGCGGAGATAACAATC | AAAGTCGCTGGCAAACTTCG |
| *Dof016275* | ACCAATAGCGAACACCTCACTGA | GGCACCTCGACGACAACCAT |
| *Dof003535* | TTTCGATCGCGACGTTCCAG | GCTTGCTCTCAGACTTTGCCA |
| *Dof014839* | TCACATCTGAAATGCCACCAAGC | CCATTTGCTTCGGGATGCTCAA |
| *Dof005734* | ATGAGTCATGCTGGAGCGAC | CCTGAAGAAGTGGGCCTAGC |
| *D0f005163* | CGGCTAAGCTTCCTAACGCT | GTGAGGCTCCTGTTTCAGGG |
| *Dof014386* | CAGGGGTGAGGGGTGTAGAA | TTTTGTCGGAAGCTCACTGGT |
| *Dof013532* | ATGACTCTCGCTACCGCCATAG | CAAGAACACGAAGAAGCCTCTCC |
| *Dof006934* | GTGAAGATAGCGAAGGCGATGG | ATGGAACACAGGCACCGTATCA |
| *Dof003361* | GTGCTGGTTGAGAAGGCGTTAC | ATGCGAGATTGCGGTGATTAAGG |
| *Dof008135* | CATCTCCGCCGATCAGAACCA | GAAGAAGCTCCTCCACGCCTT |
| *Dof003845* | CCTCCAAGTCCAGGACAACATCT | TCCATGACTCGGCTCGCTTAC |
| *Dof022187* | TCAGGCGGAGGACATAATCAGAG | AGCGTCTTCCTTGTAGAGTCCAG |
| *Dof002881* | TTGACAGTGGTATCGGCATGACT | TCCGCAACCAGATAAGCAGAGT |
| *Dof014177* | CCTTCTCAGACCTGCTTTGTG | ATGGCGAAGTAAGAGGAAGGAGA |
| *Dof013141* | AGCGAGGAGGAGATGGAGCT | GGAGATAGTTCAGCCACCGAAG |
| *GAPDH* | TTCGGAAGGATTGGAAGGCTTGTAG | GAGATGATAACCTTCTTGGCACCGC |

| Table S11 PPI networks of HSP81, WRKY33, and MYB78 proteins in D. officinale | | |
| --- | --- | --- |
| **node1** | **node2** | **combined_score** |
| BHLH94 | MYB78 | 0.169 |
| BHLH96 | MYB78 | 0.169 |
| BHLH96-2 | MYB78 | 0.169 |
| BHLH96-3 | MYB78 | 0.169 |
| BHLH96-5 | MYB78 | 0.169 |
| COR413PM2 | LTI65 | 0.596 |
| COR413PM2 | LTI65-2 | 0.596 |
| COR413PM2 | WRKY33 | 0.521 |
| DPBF3-2 | MYB78 | 0.515 |
| DPBF3-3 | MYB78 | 0.515 |
| HSP81 | PBS1 | 0.588 |
| HSP81 | PBS1-2 | 0.588 |
| HSP81 | PBS1-3 | 0.588 |
| HSP81 | PBS1-5 | 0.588 |
| HSP81 | PBS1-6 | 0.588 |
| HSP81 | WRKY33 | 0.515 |
| HSP81 | PBS1-8 | 0.588 |
| HSP81 | PBS1-9 | 0.588 |
| HSP81 | PBS1-7 | 0.588 |
| HSP81 | PBS1-12 | 0.588 |
| LTI65 | MYB78 | 0.175 |
| LTI65 | WRKY33 | 0.51 |
| LTI65 | COR413PM2 | 0.596 |
| LTI65-2 | MYB78 | 0.175 |
| LTI65-2 | WRKY33 | 0.51 |
| LTI65-2 | COR413PM2 | 0.596 |
| MYB78 | BHLH96 | 0.169 |
| MYB78 | LTI65 | 0.175 |
| MYB78 | LTI65-2 | 0.175 |
| MYB78 | BHLH94 | 0.169 |
| MYB78 | BHLH96-2 | 0.169 |
| MYB78 | BHLH96-5 | 0.169 |
| MYB78 | BHLH96-3 | 0.169 |
| MYB78 | DPBF3-3 | 0.515 |
| MYB78 | DPBF3-2 | 0.515 |
| PBS1 | WRKY33 | 0.515 |
| PBS1 | HSP81 | 0.588 |
| PBS1-12 | HSP81 | 0.588 |
| PBS1-12 | WRKY33 | 0.515 |
| PBS1-2 | WRKY33 | 0.515 |
| PBS1-2 | HSP81 | 0.588 |
| PBS1-3 | WRKY33 | 0.515 |
| PBS1-3 | HSP81 | 0.588 |
| PBS1-5 | WRKY33 | 0.515 |
| PBS1-5 | HSP81 | 0.588 |
| PBS1-6 | WRKY33 | 0.515 |
| PBS1-6 | HSP81 | 0.588 |
| PBS1-6 | PBS1-7 | 0.901 |
| PBS1-7 | PBS1-6 | 0.901 |
| PBS1-7 | HSP81 | 0.588 |
| PBS1-7 | WRKY33 | 0.515 |
| PBS1-8 | HSP81 | 0.588 |
| PBS1-8 | WRKY33 | 0.515 |
| PBS1-9 | HSP81 | 0.588 |
| PBS1-9 | WRKY33 | 0.515 |
| SIB1 | WRKY33 | 0.48 |
| WRKY33 | PBS1 | 0.515 |
| WRKY33 | PBS1-2 | 0.515 |
| WRKY33 | LTI65 | 0.51 |
| WRKY33 | LTI65-2 | 0.51 |
| WRKY33 | PBS1-3 | 0.515 |
| WRKY33 | PBS1-5 | 0.515 |
| WRKY33 | COR413PM2 | 0.521 |
| WRKY33 | PBS1-6 | 0.515 |
| WRKY33 | HSP81 | 0.515 |
| WRKY33 | PBS1-7 | 0.515 |
| WRKY33 | SIB1 | 0.48 |
| WRKY33 | PBS1-8 | 0.515 |
| WRKY33 | PBS1-9 | 0.515 |
| WRKY33 | PBS1-12 | 0.515 |

**Figure S1.** Protoplasts isolated from the stems of *D. officinale* in CK, 12hpi, and 24hpi samples.

**Figure S2.** The UMAP plot shows the expression of representative cluster-specific marker genes in the stem base of *D. officinale*.

**Figure S3.** The UMAP plot shows the expression of representative differentially expressed transcription factor genes in the stem base of *D. officinale*.

**Figure S4.** WGCNA of *D. officinale* genes in *S. rolfsii* treatment
